# Glycosylated BODIPY- Incorporated Pt(II) Metallacycles for Targeted and Synergistic Chemo-Photodynamic Therapy

**DOI:** 10.1021/acs.jmedchem.2c01940

**Published:** 2023-02-21

**Authors:** Gonzalo Durán-Sampedro, Evelyn Y. Xue, Marta Moreno-Simoni, Celia Paramio, Tomás Torres, Dennis K. P. Ng, Gema de la Torre

**Affiliations:** † Department of Organic Chemistry, Universidad Autónoma de Madrid, Campus de Cantoblanco, Madrid 28049, Spain; ‡ Institute for Advanced Research in Chemical Sciences (IAdChem), Universidad Autónoma de Madrid, Campus de Cantoblanco, Madrid 28049, Spain; § Department of Chemistry, The Chinese University of Hong Kong, Shatin, N.T., Hong Kong, China; ∥ IMDEA Nanociencia, C/Faraday 9, Cantoblanco, Madrid 28049, Spain

## Abstract

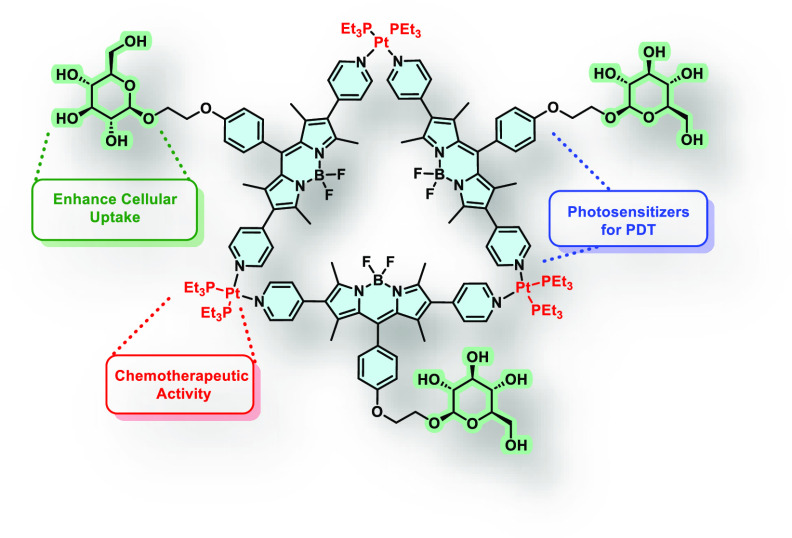

Pt(II)-BODIPY complexes combine the chemotherapeutic activity of Pt(II) with the photocytotoxicity of BODIPYs. Additional conjugation with targeting ligands can boost the uptake by cancer cells that overexpress the corresponding receptors. We describe two Pt(II) triangles, **1** and **2**, built with pyridyl BODIPYs functionalized with glucose (**3**) or triethylene glycol methyl ether (**4**), respectively. Both **1** and **2** showed higher singlet oxygen quantum yields than **3** and **4**, due to the enhanced singlet-to-triplet intersystem crossing. To evaluate the targeting effect of the glycosylated derivative, in vitro experiments were performed using glucose transporter 1 (GLUT1)-positive HT29 and A549 cancer cells, and noncancerous HEK293 cells as control. Both **1** and **2** showed higher cellular uptake than **3** and **4**. Specifically, **1** was selective and highly cytotoxic toward HT29 and A549 cells. The synergistic chemo- and photodynamic behavior of the metallacycles was also confirmed. Notably, **1** exhibited superior efficacy toward the cisplatin-resistant R-HepG2 cells.

## Introduction

Photodynamic therapy (PDT) is a noninvasive form of phototherapy that utilizes harmless light to activate non- or minimally toxic photosensitive chemicals called photosensitizers (PS) to generate cytotoxic reactive oxygen species for malignant cell eradication.^1,2^ PDT has received tremendous attention in the past decades due to its broad applicability (both to localized tumors and infections) and high selectivity and spatio-temporal resolution (only works under local illumination to the lesions). Moreover, its mode of action usually does not cause the emergence of resistance. Owing to their singular photochemical and structural characteristics, porphyrinoid-based derivatives^3−6^ have been extensively studied as PS for PDT in clinical and preclinical studies, and some of them (e.g., Visudyne and Photofrin) have already been approved by the U.S. Food and Drug Administration (FDA) for their use in cancer therapy.^7^ Several synthetic porphyrinoids (commonly called second-generation PS), which include synthetic porphyrins,^8,9^ phthalocyanines,^10−12^ and boron dipyrromethene (BODIPY) derivatives^13,14^ have been developed as PS over the years. Some of them have already been approved for the treatment of certain cancers or clinical trials. In particular, BODIPY derivatives,^15^ whose structure resembles half of a porphyrin ring, are well recognized for their excellent fluorescence properties, which are commonly employed in the context of bioimaging.^16^ BODIPYs also exhibit many desired characteristics, including strong and tunable absorption in the visible to near-infrared region, resistance to photobleaching, and high light–dark toxicity ratio. However, for their use as PS for PDT,^17,18^ they are usually modified with heavy atoms, such as halogens or transition metals, to enhance the singlet-to-triplet intersystem crossing.^13^ Conjugation of BODIPYs with transition metals usually results in weaker fluorescence quenching compared to substitution with halogen atoms,^19^ which makes BODIPY-conjugated metal complexes ideal theranostic agents for PDT and cell imaging.^20−22^ In particular Pt(II)-BODIPY metallo-supramolecular complexes have recently emerged as promising antitumoral agents,^23−25^ combining the chemotherapeutic activity of Pt(II) complexes with the photocytotoxic effect of BODIPYs.^26^ In 2018, Zhou et al. reported two two-dimensional triangles constructed with a bis(pyridyl) BODIPY as a donor ligand and two acceptor Pt(II) nodes, which exhibited a synergistic anticancer effect.^27^ These supramolecular coordination complexes were also found to be effective toward a cisplatin-resistant cell line. During the course of our investigation, Lin et al. reported the preparation of a phenylthiol-based supramolecular Pt(II)-metallacycle with red fluorescence emission and the encapsulation of this complex into nanoparticles using an amphiphilic liposome to render it biocompatible.^28^ However, all of these BODIPY-containing metallocycles are not conjugated with tumor-targeting ligands such as antibodies, peptides, and carbohydrates that can promote active uptake by cancer cells with the corresponding receptors on the surface.^6,29,30^

Among the 14 human glucose transporters (GLUTs) reported so far, glucose transporter 1 (GLUT1) is the most common member, which is overexpressed in a wide range of cancer cells, and it is responsible for their augmented glucose uptake and metabolism.^31^ Hence, GLUT1 has been exploited as an important target for the delivery of theranostic agents against cancer.^32^ Therefore, glucose was chosen to conjugate with the triangular skeleton of BODIPY-Pt(II) complexes with a view to promoting cellular uptake via the Warburg effect.^33^ Moreover, linking a biological vector such as glucose to this type of metallacycles can enhance their amphiphilic character, yielding metallo-supramolecular amphiphiles^19,34^ that can spontaneously self-assemble in aqueous solutions,^8,35^ avoiding the use of additional delivery components.

**Figure 1:**
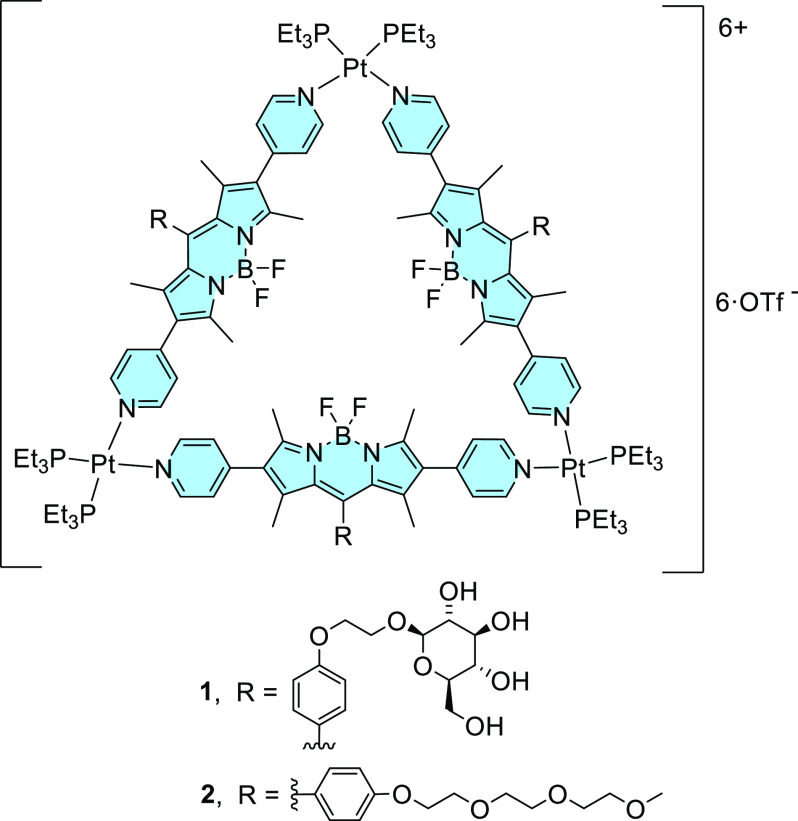
Molecular structure of metallo-supramolecular complexes **1** and **2**.

We report herein two Pt(II)-linked metallo-supramolecular amphiphiles **1** and **2**, in which the pyridyl-functionalized BODIPY ligands are further substituted with a glucose or a triethylene glycol (TEG) methyl ether moiety (Figure 1). Both metallacycles form stable nanoparticles with a uniform nanosized distribution in biological media. Detailed in vitro studies showed that they exhibited synergistic chemotherapeutic and PDT effects. The glycoconjugated analogue **1** also showed a cell-selective property and could maintain its high photocytotoxicity against a drug-resistant cancer cell line.

## Results and Discussion

### Synthesis and Characterization

To prepare the target metallo-macrocycles **1** and **2**, a 1,3,5,7-tetramethyl BODIPY skeleton was chosen due to its ease of preparation. The characteristic reactivity at the 2 and 6 positions toward electrophilic aromatic substitution was exploited to prepare a diiodo intermediate that could undergo a Suzuki coupling reaction to attach two pyridine rings. These terminal ligands were then reacted with Pt(II) complexes to form the corresponding metallo-macrocycles. To enhance the water solubility and uptake by the target cancer cells, a glucose moiety was also introduced to the BODIPY core via Williamson reaction at the phenol group attached to the *meso* position. For comparison, the TEG analogue **2** was also prepared by replacing the glucose moiety with a TEG chain.

**Scheme 1:**
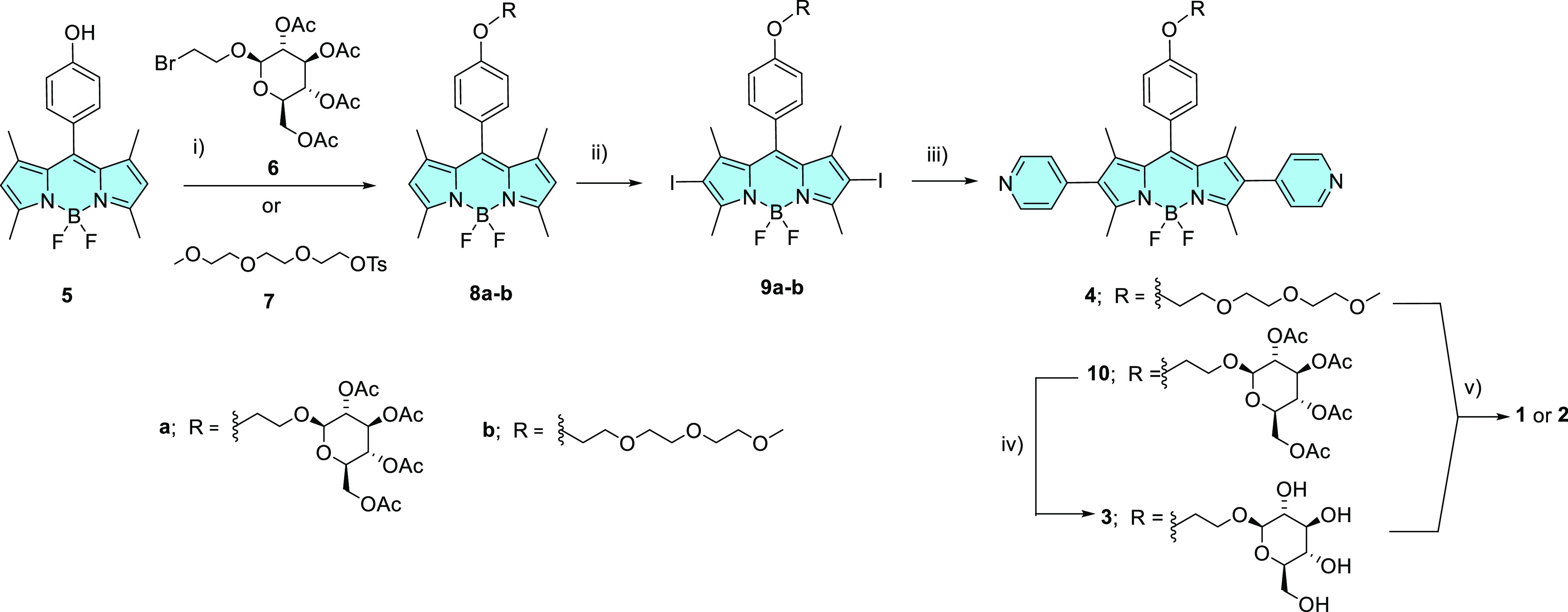
Synthesis of Pt(II)-BODIPY-Based Metallacycles **1** and **2** Conditions: (i) K_2_CO_3_, DMF, 60 °C, 16 h, 70% for **8a**; K_2_CO_3_, acetone, 70 °C, 5 h for **8b**; (ii) ICI, CH_2_Cl_2_/MeOH, rt, 10 min, 83% for **9a**; (iii) 4-pyridylboronic acid, Cs_2_CO_3_, Pd(PPh_3_)_4_, dioxane/H_2_O, 110 °C, 2 h, 33% (or 19% overall yield from **5**) for **10** and 29% overall yield from **5** for **4**; (iv) NaOMe, MeOH, 0 °C, 20 min, 62%; (v) Pt(PEt_3_)_2_(OTf)_2_, CH_2_Cl_2_/MeCN (4:1), 60 °C, 24 h, 73% for **1** and 70% for **2**.

The synthetic route used to prepare the BODIPY building blocks **3** and **4** is shown in Scheme 1. For the preparation of the glycosylated analogue **3**, the previously reported BODIPY **5**(36) was treated with the brominated derivative of β-d-glucose pentaacetate **6**,^37^ followed by iodination at the 2 and 6 positions using iodine monochloride (ICI) to yield **9a** in high yield. Suzuki coupling of **9a** with 4-pyridylboronic acid using Pd(PPh_3_)_4_ as the catalyst and Cs_2_CO_3_ as the base in a dioxane/water mixture afforded **10**, which was then subjected to alkaline hydrolysis to remove the acetate protecting groups to give the target compound **3**. Similarly, the synthesis of the TEG analogue **4** involved *O*-alkylation of **5** with monotosylated TEG monomethyl ether (**7**), followed by iodination and Suzuki coupling with 4-pyridyl boronic acid. Although the synthesis of **4** proceeded efficiently and in high overall yield, the intermediates **8b** and **9b** could not be completely purified either by column chromatography on silica gel or size-exclusion chromatography on Bio-Beads S-X1 beads. Nevertheless, the target BODIPY **4** could be isolated as a pure compound.

These BODIPY building blocks were then used to prepare the metallacycles **1** and **2** according to the previously described procedure for the preparation of related metallacycles.^27^ Compound **3** or **4** was treated with a stoichiometric amount of Pt(PEt_3_)_2_(OTf)_2_ (**11**) in CH_2_Cl_2_/MeCN (4:1 v/v) in a sealed tube at 60 °C for 24 h. The resulting supramolecular complexes were isolated by the addition of diethyl ether to induce precipitation, followed by filtration, and drying in vacuo. The successful formation of the metallacycles was confirmed by a combination of spectroscopic techniques. In the ^1^H NMR spectra in CD_3_OD, we could observe the high-field shift of the signals of the pyridine protons due to Pt(II) coordination, as well as the signals corresponding to the Et groups at the phosphine ligands. In the ^19^F NMR spectra, two signals were discernible, corresponding to the triflate anions and the F ligands attached to the boron center. In addition, a signal was observed in the ^31^P NMR spectra assignable to the PEt_3_ ligands. Strong evidence of the formation of a unique discrete metallo-supramolecular assembly was obtained from the diffusion-ordered spectroscopy (DOSY) measurements, in which the same diffusion coefficient *D* value was observed for all the resonance peaks of both **1** and **2**. Considering the linear skeleton of the starting BODIPY ligands **3** and **4**, and the coordination geometry of the Pt(II) center, the formation of either triangular or square metallacycles is plausible.^20^ However, electrospray ionization (ESI) mass spectrometry confirmed the trimeric character of the metallacycles. For both **1** and **2**, we could observe the signal corresponding to the dicationic molecular ion [M-2OTf]^2+^ at *m*/*z* = 1914.5389 and 1905.5845 for **1** and **2**, respectively, of which the isotopic pattern was in good agreement with the simulated one for the corresponding triangular metallacycle.

### Studies of the Photophysical and Aggregation Properties

**Figure 2:**
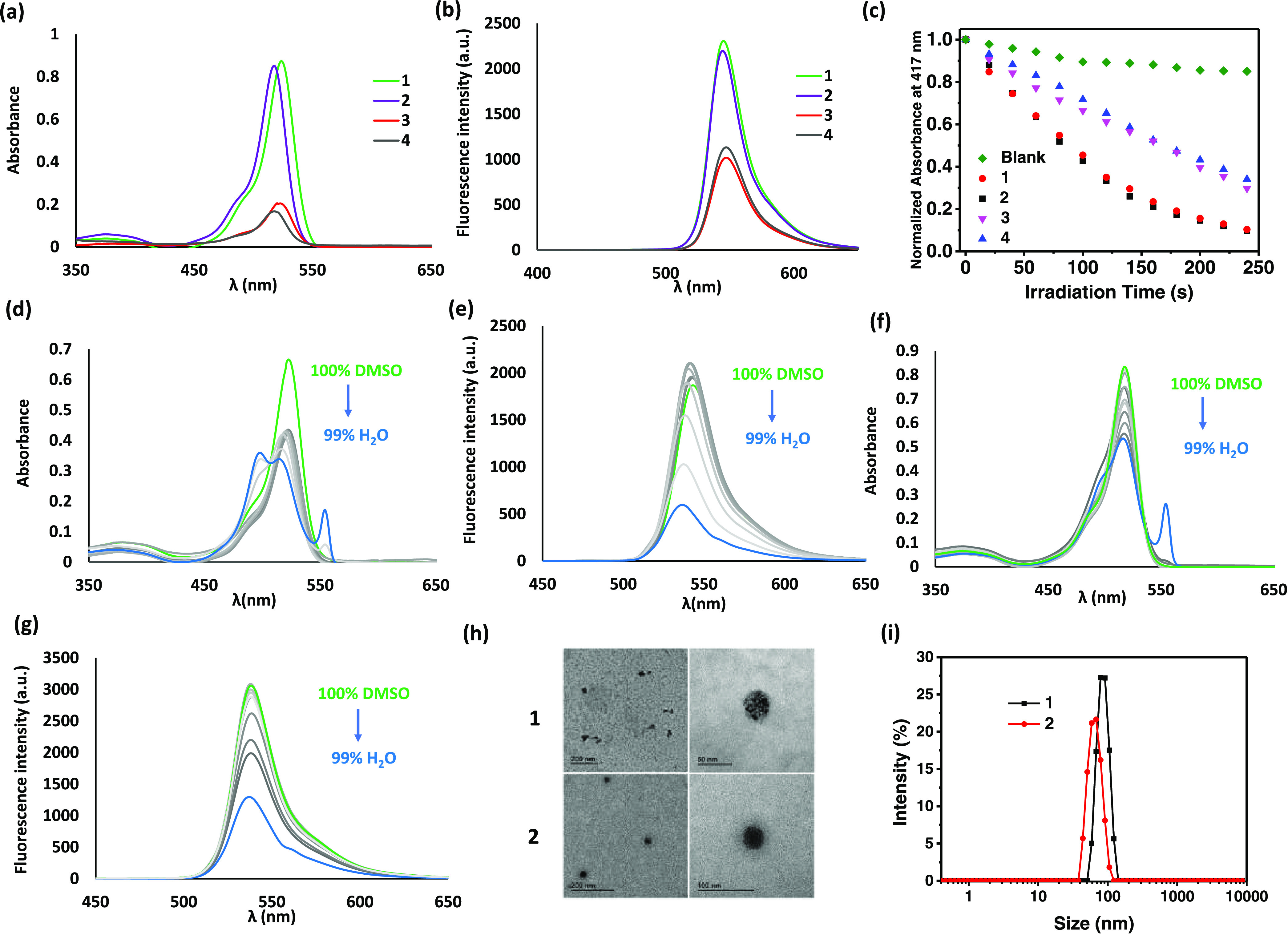
(a) Absorption and (b) fluorescence spectra of **1–4** in DMSO (2 μM). (c) Rates of decay of DPBF (initial concentration = 30 μM), as monitored spectroscopically at 417 nm, in the absence and presence of **1** (4 μM), **2** (4 μM), **3** (12 μM), or **4** (12 μM) in DMSO upon irradiation (λ = 400–700 nm). Change in (d) absorption and (e) fluorescence spectra of **1** by changing the solvent from DMSO to 1% DMSO in water. Change in (f) absorption and (g) fluorescence spectra of **2** by changing the solvent from DMSO to 1% DMSO in water. (h) TEM images of Milli-Q water solutions of **1** and **2** deposited over formvar/carbon, copper grids after glow discharge treatment. (i) Hydrodynamic diameter distribution of **1** and **2** in water.

The electronic absorption and fluorescence spectra of **1** and **2** were recorded in dimethylsulfoxide (DMSO) and compared with those of the monomeric BODIPYs **3** and **4**. The molar absorptivity of the π–π* transition of **1** and **2** was much larger than that of the corresponding precursors **3** and **4**, respectively, due to the presence of three BODIPY units in the structure (Figure 2a). The metallacycles also exhibited more intense emission than the monomeric BODIPYs (Figure 2b), but the difference in fluorescence intensity for **1**/**3** and **2/4** was not as large as the difference in absorbance. Plausibly, the fluorescence of the BODIPY components in **1** and **2** was diminished in comparison to that of **3** and **4** as a consequence of the heavy-atom effect of the Pt(II) centers, which would enhance the singlet-to-triplet intersystem crossing. To verify it, we determined and compared the singlet oxygen generation efficiency of **1**–**4** using 1,3-diphenylisobenzofuran (DPBF) as the singlet oxygen scavenger.^38^ The rate of conversion of this probe to 1,2-dibenzoylbenzene through an unstable peroxide intermediate was measured by monitoring the change in the absorbance at 417 nm of DPBF along with the irradiation time. As shown in Figure 2c, all of the compounds could efficiently consume DPBF via sensitizing the formation of singlet oxygen upon irradiation (λ = 400–700 nm), while in the absence of **1**–**4**, the absorbance of DPBF remained almost unchanged. Metallacycles **1** and **2** showed a higher singlet oxygen quantum yield (Φ_Δ_) than **3** and **4**, respectively (Table 1), as a result of the enhanced singlet-to-triplet intersystem crossing induced by the Pt(II) centers. The singlet oxygen generation efficiency of **1–4** in water was also determined and compared using 9,10-anthrancenediyl-bis(methylene)dimalonic acid (ABDA) as a water-soluble singlet oxygen probe.^38^ In this solvent, all the compounds could also effectively consume the probe upon light irradiation, and again the rate was slower for the monomeric BODIPYs **3** and **4** (Figure S1). The trend of singlet oxygen quantum yields was in good agreement with that obtained in DMSO, indicating that the singlet oxygen generation ability of **1-4** will not be quenched in biological media.

**Table 1:** Singlet Oxygen Quantum Yields of Compounds **1–4** in DMSO and H_2_O

compound	1	2	3	4
Φ_Δ_^a^	0.21	0.22	0.13	0.14
Φ_Δ_^b^	0.31	0.32	0.21	0.24

a Relative to methylene blue (Φ_Δ_ = 0.49 in DMSO) using DPBF as the singlet oxygen scavenger.^39^

b Relative to methylene blue (Φ_Δ_ = 0.52 in H_2_O) using ABDA as the singlet oxygen scavenger.^40^

To evaluate the self-assembly behavior of **1** and **2** in aqueous media, we monitored the change in the electronic absorption and fluorescence spectra of **1** and **2** in DMSO upon addition of water (Figure 2d–g). For both metallacycles, the major absorption band was diminished, broadened, and blue-shifted when the water content was increased, particularly for **1**, which indicated that the dye molecules were stacked forming *H*-aggregates. In a very high water content (e.g., 1% DMSO in water), a new absorption peak at ca. 550 nm was observed, which might suggest the co-existence of *J*-aggregates. The fluorescence band was also diminished upon addition of water for both metallacycles, but the emission band was not completely vanished even in such a high water content. All these results indicated that both **1** and **2** became aggregated in aqueous media, but they were still photophysically active, as demonstrated by their ability to generate singlet oxygen in water (Table 1). On the other hand, the monomeric analogues **3** and **4** did not show significant aggregation in aqueous media (Figure S2).

To reveal whether **1** and **2** form nanoparticles in water, we studied the aggregates of these compounds using transmission electron microscopy (TEM) and dynamic light scattering (DLS). As shown by TEM (Figure 2h), the nanoparticles of both compounds were nearly spherical in shape with a diameter of ca. 50 nm. DLS experiments rendered intensity-averaged hydrodynamic diameters of 89.0 ± 8.6 nm (for **1**) and 66.8 ± 5.1 nm (for **2**) (Figure 2i). The polydispersity indices (PDI) of **1** and **2** were determined to be 0.41 ± 0.09 and 0.36 ± 0.05, respectively. The relatively small PDI indicated that these nanoparticles were well dispersed in water without significant bundling.

Due to the supramolecular character of metallacycles **1** and **2**, their stability in biological media was then studied. We first prepared solutions of these compounds in Roswell Park Memorial Institute (RPMI) 1640 medium, and the solutions were left at 37 °C for a period of 24 h. During the course, high-performance liquid chromatography (HPLC) was used for monitoring. As shown in Figure S3, no additional signals appeared in the chromatograms of both compounds, showing that their supramolecular structure remained intact in biological media.

### Biological Assays

**Figure 3:**
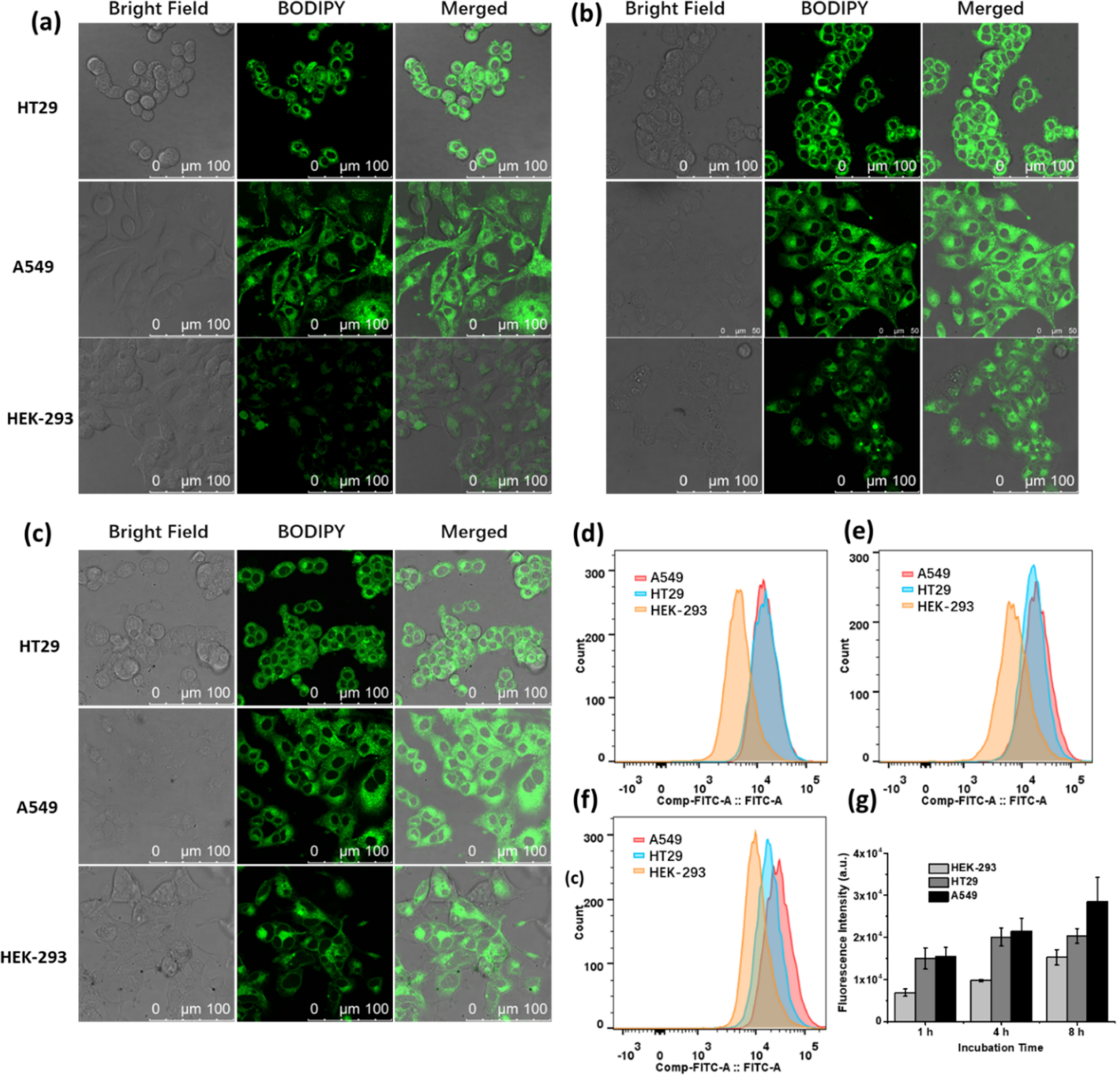
Bright field, fluorescence, and merged confocal images of HT29, A549, and HEK-293 cells after incubation with **1** (4 μM) for (a) 1 h, (b) 4 h, and (c) 8 h, respectively. (d–f) Fluorescence intensity profiles of the cells being treated under these conditions, respectively, determined by flow cytometry. (g) Corresponding quantified intracellular fluorescence intensities. Data are expressed as the mean ± standard deviation (SD) of three independent experiments.

As mentioned in the Introduction, GLUT1 has been exploited as an important target for the delivery of theranostic agents against cancer.^32^ To examine the targeting effect of the glucose moieties in the triangular Pt(II)-BODIPY complex **1**, the cellular uptake of this metallacycle was studied using the GLUT1-positive HT29 human colorectal adenocarcinoma cells and A549 human lung carcinoma cells, as well as the non-cancerous human embryonic kidney cells HEK293 used as the negative control.^41,42^ These cells were incubated with **1** (4 μM) for 1, 4, and 8 h, respectively, and then examined using confocal fluorescence microscopy. As shown in Figure 3a, bright green fluorescence due to **1** was observed in HT29 and A549 cells, while the fluorescence was much weaker in HEK293 cells after incubation for 1 h. The intensity was generally increased slightly upon prolonged incubation (to 4 and 8 h) for all of the three cell lines (Figure 3b,c, respectively). Under all these incubation conditions, the intracellular fluorescence of HEK293 cells was significantly weaker than that of the other two cell lines. Similar results were obtained by flow cytometry (Figure 3d–f). As shown in the summarized data in Figure 3g, the intracellular fluorescence intensities of **1** in HT29 and A549 cells were up to 2-fold higher than those in HEK293 cells, indicating that the uptake of **1** was generally higher toward the cancer cells than the non-cancerous cells.

To reveal whether GLUT1 was involved in the uptake of **1** by HT29 and A549 cells, a competition assay was performed using free glucose as a competitor. During the incubation of the cells with **1** (4 μM) for 1 h, glucose [2 mM (500 equiv) or 40 mM (10 000 equiv)] was added for co-incubation. The intracellular fluorescence intensities were then studied using confocal microscopy and flow cytometry. As shown in Figure S4, the intracellular fluorescence intensity of the cells was greatly reduced upon addition of glucose, and the intensity was generally decreased with increasing the concentration of glucose for both the cell lines. In the presence of 40 mM of glucose, the intracellular fluorescence intensity was reduced by ca. 60% (Figure S4d). These results indicated that the transport of **1** into the cancer cells was competitively inhibited by glucose, and this competitive inhibitory effect was in a concentration-dependent manner.

For comparison, the cellular uptake of the non-glycosylated analogue **2** was also examined against these three cell lines. It was found that the intracellular fluorescence intensities as determined by confocal microscopy and flow cytometry were not remarkably different for all of the three cell lines and for all the incubation times (1, 4, and 8 h) we used (Figure S5). It seems that the three TEG chains of **2** could greatly promote the cellular uptake of the triangular core, and even incubation for 1 h, it could saturate the cellular uptake. These chains, however, could not differentiate the cancerous and non-cancerous cells, which indirectly demonstrated the targeting role of the glucose moieties in **1**.

**Figure 4:**
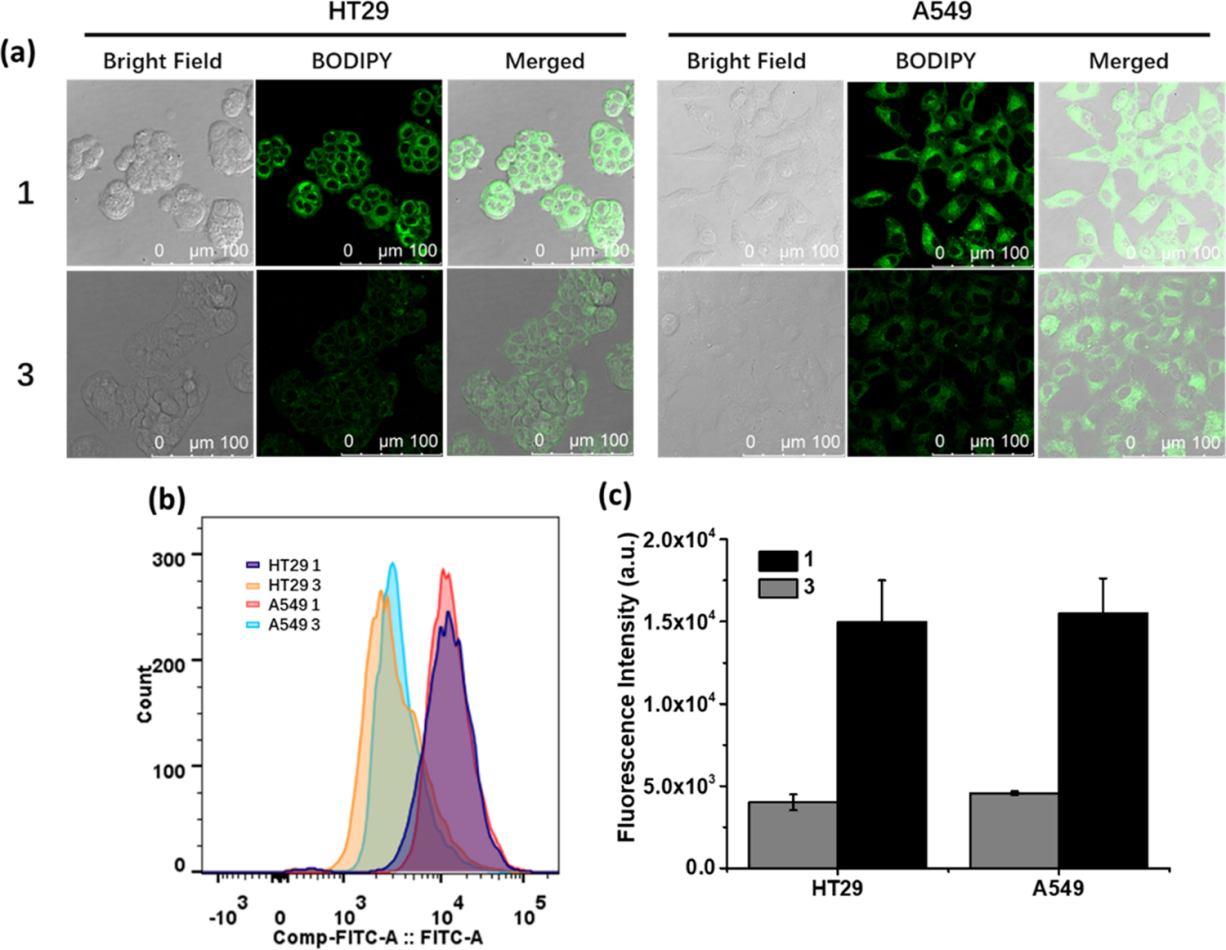
(a) Bright field, fluorescence, and merged confocal images of HT29 and A549 cells after incubation with **1** (4 μM) or **3** (12 μM) for 1 h. (b) Fluorescence intensity profiles of the cells being treated under these conditions determined by flow cytometry. (c) Corresponding quantified intracellular fluorescence intensities. Data are expressed as the mean ± SD of three independent experiments.

The cellular uptake of **1** was further compared with that of BODIPY **3** at the same concentration of the BODIPY unit. Figure 4a shows the confocal images of HT29 and A549 cells after incubation with **1** (4 μM) or **3** (12 μM) for 1 h. It was found that the fluorescence intensity of the cells was significantly stronger when they were incubated with **1** than with **3**. Flow cytometric study showed that the intensity for the former was about 3-fold of that for the latter for both cell lines (Figure 4b,c), showing that the assembled trimeric complex exhibited much higher cellular uptake than the monomeric component. Similar results have been observed for the trimeric bis(alkynyl) BODIPY-Pt(II) analogues^27^ and dipyridyl [1,2,5]thiadiazolo[3,4-*f*]benzotriazole-Pt(II) metallacycles^43^ reported previously.

The subcellular localization of **1** in A549 cells was also investigated. After incubation with **1** (4 μM) for 1 h, the cells were stained with LysoTracker Deep Red (0.1 μM for 30 min), MitoTracker Red CMXRos (0.1 μM for 20 min), or ER-Tracker Red (1 μM for 20 min), and subsequently analyzed by confocal fluorescence microscopy (Figure S6). It was found that the intracellular fluorescence of **1** could only be overlapped with that of LysoTracker, which suggested that **1** was mainly localized in the lysosomes. It is likely that, due to the presence of tumor-targeting glucose moieties, the compound was internalized through receptor-mediated endocytosis and was eventually localized in the lysosomes, which are the last compartments of the endocytic pathway.

**Figure 5:**
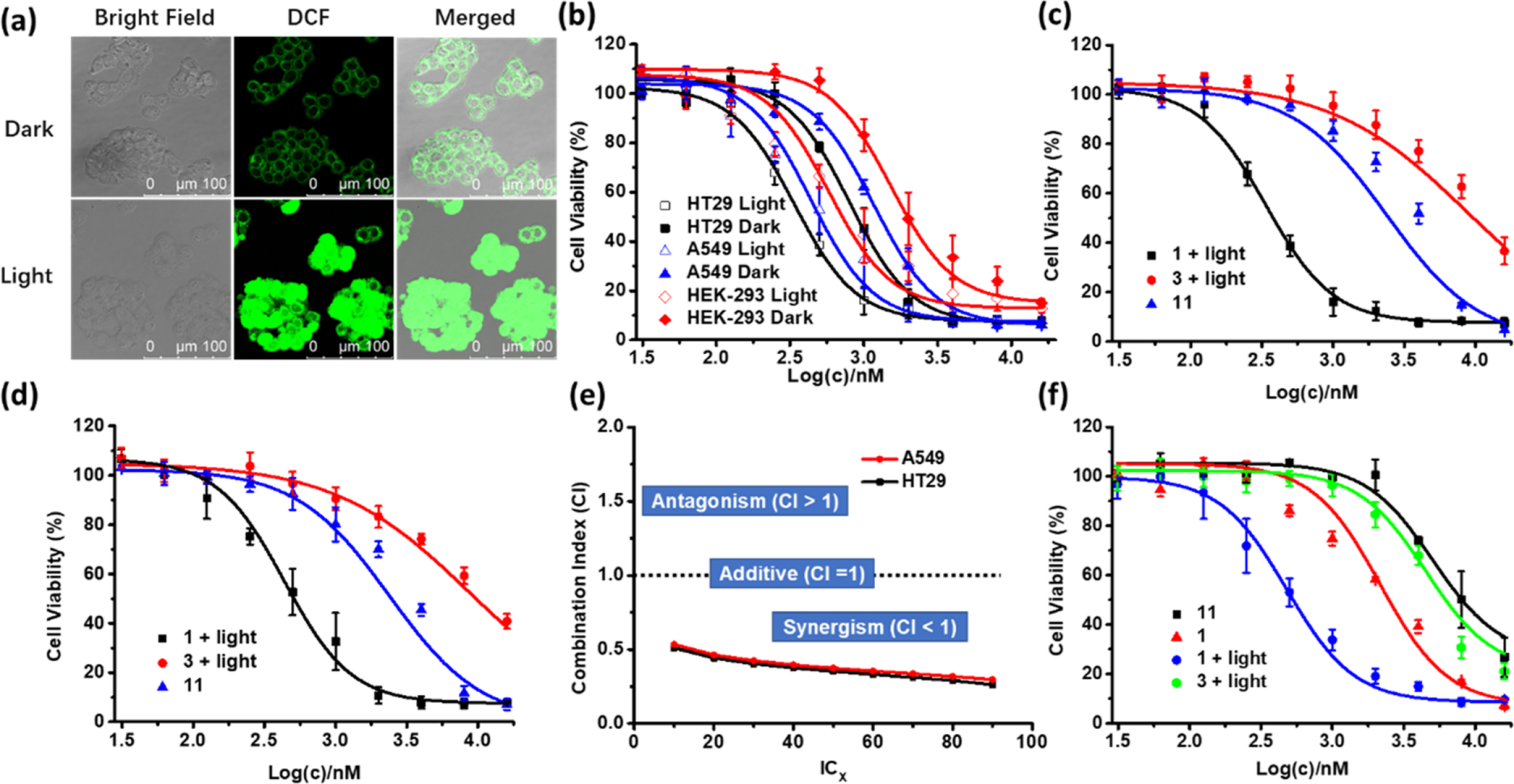
(a) Intracellular ROS generation induced by **1** (4 μM) in HT29 cells, as reflected by the fluorescence of DCF in the absence and presence of light (λ = 400–700 nm, 23 mW cm^–2^, 28 J cm^–2^). (b) Comparison of the cytotoxic effect of **1** against HT29, A549, and HEK-293 cells in the absence and presence of light (λ = 400–700 nm, 23 mW cm^–2^, 28 J cm^–2^). Comparison of the cytotoxic effect of **1**, **3**, and **11** against (c) HT29 and (d) A549 cells. For the treatment with **1** and **3**, light irradiation (λ = 400–700 nm, 23 mW cm^–2^, 28 J cm^–2^) was also applied. The concentrations for **3** and **11** were multiplied by 3 in the figures. (e) Variation of the combination index with the IC value determined from the dose-dependent survival curves of **1**, **3**, and **11** against HT29 and A549 cells. (f) Comparison of the cytotoxic effect of **1**, **3**, and **11** against the chemo-resistant R-HepG2 cells in the absence and presence of light (λ = 400–700 nm, 23 mW cm^–2^, 28 J cm^–2^). For (b)–(d) and (f), data are expressed as the mean ± standard error of the mean (SEM) of three independent experiments, each performed in quadruplicate.

The singlet oxygen generation efficiency of **1** in HT29 cells was then evaluated using 2′,7′-dichlorodihydrofluorescein diacetate (H_2_DCFDA) as the singlet oxygen probe.^44^ The cells were first incubated with **1** (4 μM) for 1 h, and then with H_2_DCFDA (50 μM) for 30 min, followed by light irradiation (λ = 400–700 nm, 23 mW cm^–2^) for 20 min or leaving in the dark for the same period of time. As shown in Figure 5a, the green fluorescence due to the oxidized product 2′,7′-dichlorofluorescein (DCF) was weak for the cells without the light treatment. In contrast, very bright fluorescence was observed for the cells with light irradiation, reflecting the high intracellular singlet oxygen generation efficiency of **1**.

The cytotoxicity of **1** against HT29, A549, and HEK293 cells was subsequently studied using the 3-(4,5-dimethylthiazol-2-yl)-2,5-diphenyltetrazolium bromide (MTT) assay. Figure 5b shows the dose-dependent survival curves of **1** for the three cell lines both with and without the light treatment (λ = 400–700 nm, 23 mW cm^–2^, 28 J cm^–2^). It can be seen that **1** was cytotoxic toward all of the three cell lines even in the dark, which could be attributed to the chemotherapeutic effect of the platinum moieties. The dark cytotoxicity against HT29 and A549 cells was significantly higher than that for HEK293 cells as a result of the higher uptake of **1** by the former two cell lines. Upon light irradiation, the cytotoxicity of **1** was generally increased for all of the three cell lines due to the photodynamic effect of the BODIPY moieties. The half-maximal inhibitory concentrations (IC_50_) for HT29, A549, and HEK293 cells were determined to be 0.42, 0.56, and 0.85 μM, respectively. It is worth noting that at these concentrations, the dark cytotoxicity of **1** was negligible for the noncancerous HEK293 cells.

Being encouraged by these promising results, we further determined whether the two cytotoxic effects work in a synergistic manner. In this study, the photocytotoxicity of **1** was compared with the photocytotoxicity of BODIPY **3** and the dark cytotoxicity of Pt(PEt_3_)_2_(OTf)_2_ (**11**) against HT29 and A549 cells (Figure 5c,d, respectively). Based on these dose-dependent survival curves, the combination indices (CI) were calculated at different IC values. In combination therapy, a CI value greater than, equal to, or lower than 1 denotes antagonism, additivity, or synergism, respectively.^45^ As shown in Figure 5e, all of the CI values at IC_10_ to IC_90_ were much lower than 1 for both the cell lines, suggesting that the photodynamic effect of the BODIPY moieties and the chemotherapeutic effect of the Pt(II) components were highly synergistic to each other for **1**.

**Figure 6:**
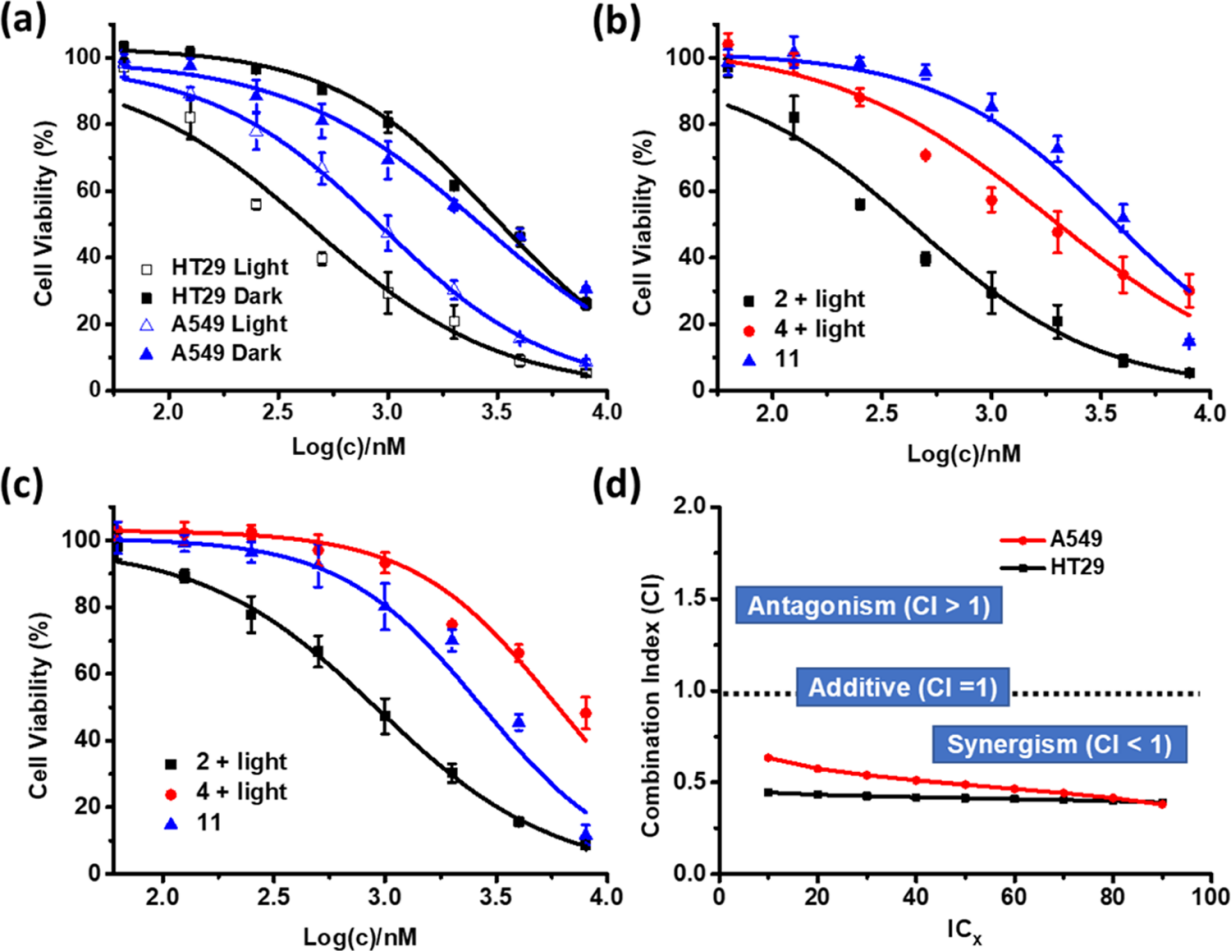
(a) Comparison of the cytotoxic effect of **2** against HT29 and A549 cells in the absence and presence of light (λ = 400–700 nm, 23 mW cm^–2^, 28 J cm^–2^). Comparison of the cytotoxic effect of **2**, **4**, and **11** against (b) HT29 and (c) A549 cells. For the treatment with **2** and **4**, light irradiation (λ = 400–700 nm, 23 mW cm^–2^, 28 J cm^–2^) was also applied. The concentrations for **4** and **11** were multiplied by 3 in the figures. For (a)–(c), data are expressed as the mean ± SEM of three independent experiments, each performed in quadruplicate. (d) Variation of the combination index with the IC value determined from the dose-dependent survival curves of **2**, **4**, and **11** against HT29 and A549 cells.

Similarly, the cytotoxicity of **2** was also examined and compared with that of the model compounds BODIPY **4** and Pt(PEt_3_)_2_(OTf)_2_ (**11**). As shown in Figure 6a, **2** was cytotoxic toward HT29 and A549 cells, and light irradiation could significantly enhance the cytotoxicity. Upon irradiation, the IC_50_ values were determined to be 0.34 μM (for HT29 cells) and 0.93 μM (for A549 cells), which were comparable with those of **1**. By comparing the cytotoxicity of **2** and **4** with light irradiation with that of **11** without light irradiation (Figure 6b,c), it was found that the CI values were also consistently lower than 1 (Figure 6d), showing that the two cytotoxic effects also worked synergistically for **2** against the two cell lines. All these results demonstrated that these supramolecular metallacycles could serve as a promising platform for synergistic chemo- and photodynamic anticancer therapy.

Drug resistance is a great challenge for chemotherapy. It has been reported that by combining this treatment modality with PDT, the therapeutic outcome can be synergistically improved and the drug resistance can also be circumvented through multipronged cell-killing pathways.^46,47^ To preliminary explore the potential of **1** in addressing the problem of drug resistance, we employed the R-HepG2 human hepatoma cells as a model cell line, which has been reported to exhibit drug resistance to a variety of functionally and structurally unrelated chemotherapeutic agents,^48^ and compared the cytotoxicities of **1**, **3**, and Pt(PEt_3_)_2_(OTf)_2_ (**11**) under different conditions. The results are summarized in Figure 5f. It can be seen that **1** showed significantly higher cytotoxicity upon light irradiation compared with the photocytotoxic effect of **3** and the dark cytotoxic effect of **1** and **11**. The IC_50_ values were determined to be 0.59, 17.8, 1.44, and 24.1 μM, respectively. It is worth noting that the IC_50_ value of **11** against R-HepG2 cells (24.1 μM) was much higher than that toward the non-drug-resistant HT29 (12.5 μM) and A549 (10.9 μM) cells. In contrast, the trimeric complex **1** showed similar photocytotoxicity (0.59 μM for R-HepG2 cells vs 0.42 and 0.56 μM for HT29 and A549 cells, respectively), indicating that it remains highly potent toward the drug-resistant cancer cells.

## Conclusions

We have prepared and fully characterized two amphiphilic BODIPY-incorporated Pt(II) metallo-supramolecular triangles which have been endowed with biocompatible glucose or TEG moieties. The enhanced and selective cellular uptake of the glycosylated analogue **1** directed by its self-assembled nanostructure in aqueous media and the tumor-directing glucose moieties, as well as the high ^1^O_2_ generation efficiency and the synergistic chemotherapeutic and PDT effects of both compounds have also been demonstrated. In particular, the glucose-substituted metallacycle **1** has emerged as a promising anticancer agent. Apart from its tumor-targeting property toward the GLUT-overexpressing cancer cells, which is not present in the previously reported Pt(II)-BODIPY metallo-supramolecular complexes, the compound exhibits high chemo- and photocytotoxicities against a range of cancer cell lines, including the cisplatin-resistant R-HepG2 cells

## Experimental Section

### General Information

All chemical reagents were of analytical grade and purchased from commercial sources and used without further purification. Anhydrous solvents were acquired by standard methods prior to use. The monitoring of reactions was carried out by thin layer chromatography (TLC), employing aluminum sheets coated with silica gel type 60 F254 (0.2 mm thick, Merck) and a UV lamp of 254 and 365 nm for visualization. Purification of the synthesized products was performed by normal-phase column chromatography using silica gel (230–400 mesh, 0.040–0.063 mm, Merck). Eluents along with the relative ratio in the case of solvent mixtures are indicated for each case. Nuclear magnetic resonance (^1^H, ^13^C, ^11^B, and ^19^F NMR) spectra were recorded on a Bruker AV-300 or a Bruker DRX-500 spectrometer. The deuterated solvent employed in each case is indicated in brackets, and its residual peak was used to calibrate the spectra using the literature reference δ ppm values. All of the spectra were recorded at room temperature. Matrix-assisted laser desorption/ionization time-of-flight (MALDI-TOF) mass spectra were taken on a Bruker-Ultraflex-III spectrometer with a Nd:YAG laser operated at 355 nm. ESI mass spectra were recorded on an API QSTAR Pulsar I mass spectrometer. Ultraviolet and visible (UV-Vis) spectra were recorded on a JASCO-V660 spectrophotometer using solvents of the spectroscopic grade. Fluorescence measurements were carried out with a JASCO-V8600 spectrofluorometer. The synthesis and characterization of **5**-**7** were reported previously.^36,37^ Compounds **1** and **2** were isolated at greater than 95% purity, as assessed by HPLC (see Supporting Information).

### General Procedure for the Synthesis of **8a**-**b**

In a round-bottom flask, **5** (1 equiv), freshly prepared **6** or **7** (5 equiv), and K_2_CO_3_ (5 equiv) were refluxed in acetonitrile (20 mL) for 16 h. After **5** was consumed as indicated by TLC, the solvent was evaporated off under vacuum. CH_2_Cl_2_ was added, and the organic phase was washed three times with water. The organic phase was dried over anhydrous MgSO_4_. After evaporation, the crude product was purified by column chromatography on silica gel using a mixture of heptane/ethyl acetate (1:1 v/v) as eluent.

BODIPY **8a**, a brown solid, yield 70%. ^1^H NMR (300 MHz, CDCl_3_): δ 7.17 (d, *J* = 7.9 Hz, 2 H), 6.99 (d, *J* = 8.0 Hz, 2 H), 5.97 (s, 2 H), 5.26–5.22 (m, 1 H), 5.13–5.09 (m, 1 H), 5.04–5.02 (m, 1 H), 4.96 (d, *J* = 8.0 Hz, 1 H), 4.29–4.26 (m, 1 H), 4.17–4.14 (m, 3 H), 4.00–3.96 (m, 1 H), 3.76–3.72 (m, 1 H), 3.69 (s, 1 H), 2.55 (s, 6 H), 2.09 (s, 3 H), 2.03 (s 3 H), 2.01 (s, 3 H), 1.98 (s, 3 H), 1.41 (s, 6 H). ^13^C NMR (126 MHz, CDCl_3_): δ 170.6, 170.3, 169.4, 169.3, 159.1, 143.0, 141.6, 131.8, 129.3, 127.5, 121.1, 115.1, 101.1, 72.7, 72.0, 71.2, 68.4, 68.0, 67.2, 67.1, 61.9, 20.7, 20.6, 20.6, 20.6, 14.6. ^19^F NMR (471 MHz, CDCl_3_): δ −146.28 (m). ^11^B NMR (160 MHz, CDCl_3_): δ 0.76 (t, *J* = 33.1 Hz). HRMS (MALDI-TOF): *m*/*z* calc for C_35_H_41_BF_2_N_2_O_11_ [M^+^]: 714.2772, found: 714.2774.

BODIPY **8b**, a brown solid.^49^^1^H NMR (300 MHz, CDCl_3_): δ 7.16 (d, *J* = 8.4 Hz, 2 H), 7.02 (d, *J* = 8.4 Hz, 2 H), 5.97 (s, 2 H), 4.19 (t, *J* = 4.8 Hz, 2 H), 3.91 (t, *J* = 4.8 Hz, 2 H), 3.76–3.79 (m, 2 H), 3.70–3.72 (m, 2 H), 3.66–3.69 (m, 2 H), 3.56–3.58 (m, 2 H), 3.39 (s, 3 H), 2.55 (s, 6 H), 1.42 (s, 6 H).

### General Procedure for the Synthesis of **9a**-**b**

To a stirred solution of **8a** or **8b** (1 equiv) in a mixture of CH_2_Cl_2_/MeOH (1:1 v/v) was added dropwise a solution of ICl (1 M in CH_2_Cl_2_, 2.5 equiv). The mixture was stirred at r.t. for 10 min. After the consumption of the starting materials as indicated by TLC, the solvent was evaporated under vacuum, and the resulting mixture dissolved in CH_2_Cl_2_. The solution was then washed with H_2_O, dried over anhydrous MgSO_4_, filtered, and concentrated to dryness. The crude product was purified by flash chromatography on silica gel using a mixture of heptane/ethyl acetate (1:1 v/v) as eluent.

BODIPY **9a**, a red solid, yield 83%. ^1^H NMR (300 MHz, CDCl_3_): δ 7.14 (d, *J* = 8.5 Hz, 2 H), 7.02 (d, *J* = 8.6 Hz, 2 H), 5.28–5.22 (m, 1 H), 5.15–5.02 (m, 2 H), 4.70 (d, *J* = 7.9 Hz, 1 H), 4.32–4.16 (m, 5 H), 4.02–3.97 (m, 1 H), 3.78–3.72 (m, 1 H), 2.63 (s, 6 H), 2.10 (s, 3 H), 2.04 (s, 3 H), 2.01 (s, 3 H), 1,99 (s, 3 H), 1.43 (s, 6 H). ^13^C NMR (75 MHz, CDCl_3_): δ 170.6, 170.3, 169.4, 169.3, 159.6, 156.7, 145.3, 141.3, 131.7, 129.2, 127.2, 115.4, 101.1, 85.6, 72.7, 72.0, 71.0, 68.4, 68.0, 67.2, 61.9, 20.8, 20.7, 20.6, 20.6, 17.2, 16.0. HRMS (MALDI-TOF): *m*/*z* calc for C_35_H_39_BF_2_I_2_N_2_NaO_11_ [M^+^]: 989.0603, found: 989.0584.

BODIPY **9b**, a red solid.^45^^1^H NMR (300 MHz, CDCl_3_): δ 7.16 (d, *J* = 8.4 Hz, 2 H), 7.02 (d, *J* = 8.4 Hz, 2 H), 4.19 (t, *J* = 4.8 Hz, 2 H), 3.91 (t, *J* = 4.8 Hz, 2 H), 3.76–3.79 (m, 2 H), 3.70–3.72 (m, 2 H), 3.66–3.69 (m, 2 H), 3.56–3.58 (m, 2 H), 3.39 (s, 3 H), 2.55 (s, 6 H), 1.42 (s, 6 H).

### General Procedure for the Suzuki Cross-Coupling Reactions

The iodinated BODIPY **9a** or **9b** (1 equiv), 4-pyridinylboronic acid (4 equiv), Cs_2_CO_3_ (4 equiv), and Pd(PPh_3_)_4_ (0.1 equiv) were mixed in a 100 mL Schlenk flask, and then degassed and backfilled with Ar three times. A degassed mixture of dioxane/water (10:1 v/v) (1 mM for **9a** or **9b**) was introduced into the reaction flask by a syringe. The mixture was heated under reflux under an inert atmosphere at 110 °C for 2 h. The solvent was then removed under reduced pressure. CH_2_Cl_2_ was added, and the solution was washed three times with water, followed by drying over anhydrous MgSO_4_. After evaporation, the crude product was purified by column chromatography on silica gel using a mixture CH_2_Cl_2_/MeOH (99:1 v/v) as eluent.

BODIPY **10**, an orange solid, yield 33%. ^1^H NMR (500 MHz, CDCl_3_): δ 8.63 (d, *J* = 5.9 Hz, 4 H), 7.24 (d, *J* = 8.7 Hz, 2 H), 7.10 (d, *J* = 6.0 Hz, 2 H), 7.04 (d, *J* = 8.7 Hz, 2 H), 5.23 (t, *J* = 9.5 Hz, 1 H), 5.10 (t, *J* = 9.7 Hz, 1 H), 5.02 (dd, *J* = 9.6, 8.0 Hz, 1 H), 4.67 (d, *J* = 8.0 Hz, 1 H), 4.27 (dd, *J* = 12.3, 4.6 Hz, 1 H), 4.19–4.15 (m, 4 H), 3.99–3.95 (m, 1 H), 3.75–3.72 (m, 1 H), 2.56 (s, 6 H), 2.07 (s, 3 H), 2.02 (s, 3 H), 2.00 (s, 3 H), 1.96 (s, 3 H), 1.38 (s, 6 H). ^13^C NMR (126 MHz, CDCl_3_): δ 170.7, 170.4, 169.5, 169.4, 159.7, 154.2, 150.0, 143.4, 142.0, 140.0, 132.1, 131.3, 129.3, 127.4, 125.1, 115.6, 101.2, 72.8, 72.1, 71.3, 68.5, 68.1, 67.4, 62.0, 20.9, 20.8, 20.7, 13.5, 13.1. ^19^F NMR (471 MHz, CDCl_3_): δ −145.88 (m). ^11^B NMR (160 MHz, CDCl_3_): δ 0.90 (t, *J* = 32.9 Hz). HRMS (MALDI-TOF): *m*/*z* calc for C_45_H_47_BF_2_N_2_O_11_ [M^+^]: 868.3305, found: 868.3316.

BODIPY **4***,* an orange solid, yield 51%. ^1^H NMR (300 MHz, CDCl_3_): δ 8.64 (d, *J* = 5.1 Hz, 4 H), 7.22 (d, *J* = 8.4 Hz, 3 H), 7.14 (d, *J* = 5.1 Hz, 4 H), 7.06 (d, *J* = 8.5 Hz, 2 H), 4.20–4.16 (m, 2 H), 3.90 (t, *J* = 4.6 Hz, 3 H), 3.75 (dd, *J* = 6.1, 3.4 Hz, 3 H), 3.70 (d, *J* = 5.4 Hz, 2 H), 3.66–3.63 (m, 2 H), 3.55 (dd, *J* = 6.0, 3.4 Hz, 3 H), 3.37 (d, *J* = 0.9 Hz, 3 H), 2.56 (s, 6 H), 1.40 (s, 6 H). ^13^C NMR (76 MHz, CDCl_3_): δ 159.9, 154.2, 149.3, 143.7, 142.7, 140.2, 131.0, 129.1, 125.3, 115.8, 72.1, 71.0, 70.8, 70.7, 69.8, 67.7, 59.2, 13.5, 13.2. HRMS (MALDI-TOF): *m*/*z* calc for C_36_H_39_BF_2_N_4_O_4_ [M^+^]: 640.3030, found: 640.3030.

### Synthesis of BODIPY **3**

BODIPY **10** (1 equiv) and NaOMe (5 equiv) were dissolved in MeOH (5 mL) at 0 °C. The mixture was stirred for 30 min and then warmed to room temperature. Once the starting material **10** was consumed as shown by TLC, Dowex 50 WX8 H+ resin was added, and the mixture was stirred until it was neutral. The resin was filtered off, and the solution was evaporated under reduced pressure to give **3** as an orange solid (62% yield). ^1^H NMR (300 MHz, D_2_O): δ 8.80 (d, *J* = 5.2 Hz, 4 H), 7.93 (d, *J* = 5.3 Hz, 4 H), 7.25–7.23 (m, 2 H), 7.17–7.16 (m, 2 H), 4.57 (d, *J* = 7.9 Hz, 2 H), 4.30–4.25 (m, 3 H), 4.09–4.06 (m 1 H), 3.89 (d, *J* = 12.4 Hz, 1 H), 3.72 (dd, *J* = 12.3, 5.6 Hz, 1 H), 3.54–3.31 (m, 3 H), 2.62 (s, 6 H), 1.51 (s, 6 H). ^13^C NMR (126 MHz, D_2_O): δ 159.4, 155.0, 152.1, 145.8, 143.0, 140.8, 132.3, 129.2, 128.8, 127.8, 126.1, 115.9, 102.5, 75.9, 75.8, 73.1, 69.6, 68.2, 67.5, 60.7, 12.9, 12.8. ^19^F-NMR (126 MHz, D_2_O): δ −142.8 (m). ^11^B NMR (160 MHz, D_2_O): δ 0.85 (t, *J* = 28.9 Hz). HRMS (MALDI-TOF): *m*/*z* calc for C_35_H_41_BF_2_N_2_O_11_ [M^+^]: 714.2772, found: 714.2774.

### General Procedure for the Synthesis of **1** and **2**

BODIPY **3** or **4** (1 equiv) and Pt(PEt_3_)_2_(OTf)_2_ (1 equiv) were placed into a 20 mL pressure tube charged with a small stir bar. A mixture of CH_2_Cl_2_/MeCN (4:1 v/v) (5 mL) was added, and then the tube was sealed followed by stirring at 60 °C for 24 h. The solvent was removed under vacuum, and diethyl ether was slowly added to precipitate the corresponding metallo-macrocyle.

**Metallo-macrocycle 1**, an orange solid, yield 73%. ^1^H NMR (300 MHz, CD_3_OD): *δ* 8.70 (d, *J* = 6.0 Hz, 12 H), 7.30 (d, *J* = 5.8 Hz, 12 H), 7.12–6.91 (m, 12 H), 5.06 (t, *J* = 9.3 Hz, 4 H), 4.83 (t, *J* = 9.8 Hz, 4 H), 4.11–3.62 (m, 24 H), 3.27 (q, *J* = 7.0 Hz, 3 H), 2.16 (s, 18 H), 1.75 (m, 36 H), 1.10 (s, 72 H). HRMS (ESI): *m*/*z* calc for C_157_H_215_B_3_F_18_N_12_O_29_P_6_Pt_3_S_4_ [M – 2OTf^–^]^2+^: 1914.5319, found 1914.5386.

**Metallo-macrocycle 2**, an orange solid, yield 70%. ^1^H NMR (300 MHz, CD_3_OD): *δ* 8.70 (d, *J* = 6.0 Hz, 12 H), 7.30 (d, *J* = 5.8 Hz, 12 H), 7.12–6.91 (m, 12 H), 5.06 (t, *J* = 9.3 Hz, 4 H), 4.83 (t, *J* = 9.8 Hz, 4 H), 4.11–3.62 (m, 24 H), 3.27 (q, *J* = 7.0 Hz, 3 H), 2.16 (s, 18 H) 1.75 (m, 36 H) 1.10 (s, 72 H). ^31^P NMR (202 MHz, acetone-*d*_6_), δ (ppm): 0.18. ^19^F NMR (471 MHz, CD_3_OD): δ −79.9, −146.0. HRMS (ESI): *m*/*z* calc for C_144_H_207_B_3_F_18_N_12_O_24_P_6_Pt_3_S_4_ [M – 2OTf^–^]^2+^: 1905.5812, found: 1905.5845.

### Preparation and Characterization of Nanoparticles of **1** and **2**

Stock solutions of **1** and **2** in DMSO (both at 1 mM) were first prepared. These solutions (100 μL) were then added dropwise in water (900 μL), respectively, followed by sonication for 1 h to give the corresponding self-assembled nanoparticles. The morphology of the nanoparticles was determined using TEM. Samples were imaged using a microscope JEOL JEM 1400 plus from ICTS - Centro Nacional de Microscopía Electrónica, UCM. Nanoparticle suspensions were diluted to 10 μM, deposited by drop-casting over formvar/carbon 200 mesh, copper FCF200-CU grids with glow-discharge treatment, and dried under air before the analysis. Hydrodynamic diameters were measured using a Malvern Panalytical Zetasizer Nano ZS90 analyzer equipped with a 4 mW 633 nm He–Ne laser. Nanoparticle suspensions were diluted to 2 μM. The temperature of the samples was allowed to be equilibrated at 25 °C for 120 s before each measurement.

### Determination of Singlet Oxygen Quantum Yields

The values of singlet oxygen quantum yields (Φ_Δ_) were calculated by using methylene blue in DMSO (Φ_Δ_ = 0.49)^39^ or H_2_O (Φ_Δ_ = 0.52)^40^ as the reference, and DPBF or ABDA as the singlet oxygen scavenger, respectively. A solution of the sample with DPBF (30 μM) in DMSO or ABDA (50 μM) in H_2_O was irradiated with red light from a 300 W halogen lamp after passing through a water tank for cooling and a color filter with a cut-on wavelength between 400 and 700 nm. The absorption maximum of DPBF at 417 nm or ABDA at 378 nm was monitored along with time. The Φ_Δ_ values were calculated according to the equation
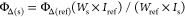
where Φ_Δ(ref)_ refers to the Φ_Δ_ of methylene blue in DMSO or H_2_O; *W*_s_ and *W*_ref_ stand for the photobleaching rates of DPBF or ABDA in the presence of the sample and the reference, respectively; and *I*_s_ and *I*_ref_ are the rates of light absorption by the sample and the reference, respectively.

### Cell Lines and Culture Conditions

HT29 human colorectal adenocarcinoma cells and R-HepG2 drug-resistant human hepatoma cells were maintained in RPMI 1640 medium (Invitrogen, no. 23400-021) supplemented with fetal bovine serum (FBS) (Thermo Fisher Scientific, cat. no. 10270-106) (10%) and penicillin-streptomycin solution (100 units mL^–1^ and 100 μg mL^–1^, respectively). A549 human lung carcinoma cells and HEK-293 human embryonic kidney cells were maintained in Dulbecco’s modified Eagle’s medium (DMEM) (Thermo Fisher Scientific, cat. no. 12100-046) supplemented with FBS (10%) and penicillin-streptomycin solution (100 units mL^–1^ and 100 μg mL^–1^, respectively). To maintain the drug resistance, R-HepG2 cells were cultured with 1.2 μM doxorubicin during passages. All of the cells were grown at 37 °C in a humidified 5% CO_2_ atmosphere.

### Study of Intracellular Fluorescence Emission

Approximately 2 × 10^5^ HT29, A549, and HEK-293 cells in the culture medium (2 mL) were seeded on glass-bottom dishes and incubated overnight at 37 °C under 5% CO_2_. The cells were treated with **1** or **2** (4 μM) for 1, 4, and 8 h, respectively. After being rinsed with phosphate-buffered saline (PBS) for three times, the cells were rinsed with Hank’s Balanced Salt Solution (HBSS) before being examined using a Leica TCS SP8 high-speed confocal microscope equipped with a 488 nm argon laser. The BODIPY unit was excited at 488 nm, and its fluorescence was monitored at 500–600 nm.

### Subcellular Localization Studies

Approximately 2 × 10^5^ A549 cells in cell culture medium (2 mL) were seeded on a confocal dish and incubated overnight at 37 °C in a humidified 5% CO_2_ atmosphere. After being rinsed with PBS, the cells were incubated with **1** (4 μM) at 37 °C for 1 h. After that, the cells were stained with LysoTracker Deep Red (Thermo Fisher Scientific, Inc., L12492) (0.1 μM for 30 min), MitoTracker Red CMXRos (Thermo Fisher Scientific, Inc., M7512) (0.1 μM for 20 min), or ER-Tracker Red (Thermo Fisher Scientific, Inc., E34250) (1 μM for 20 min) in a serum-free medium at 37 °C. The solutions were then removed, and the cells were rinsed with PBS twice before being examined with a Leica TCS SP8 high-speed confocal microscope equipped with a 488 nm laser, a 552 nm laser, and a 638 nm laser. LysoTracker Deep Red was excited at 638nm, and the fluorescence was monitored at 650–680 nm. MitoTracker Red CMXRos and ER-Tracker Red were excited at 552 nm, and their fluorescence was monitored at 590–620 nm. Compound **1** was excited at 488 nm and its fluorescence was monitored at 500–600 nm. The images were digitized and analyzed using a Leica Application Suite X software.

### Competition Assay

Approximately 2 × 10^5^ HT29 and A549 cells in the culture medium (2 mL) were incubated on a glass-bottom confocal dish overnight at 37 °C in a humidified 5% CO_2_ atmosphere. After removal of the medium, the cells were rinsed with PBS and incubated with **1** (4 μM) with or without co-incubation with free d-glucose at different concentrations (2 and 40 mM) at 37 °C for 1 h. After removal of the medium and being rinsed with PBS twice, the cells were replenished with 1 mL of HBSS before being examined using a Leica TCS SP8 high-speed confocal microscope equipped with a 488 nm argon laser. The fluorescence was monitored at 500–600 nm.

### Study of Intracellular Singlet Oxygen Generation

Approximately 2 × 10^5^ HT29 cells on glass-bottom dishes were first treated with **1** (4 μM) at 37 °C for 1 h. After being rinsed with PBS for three times, the cells were incubated with H_2_DCFDA (50 μM) for 30 min, followed by washing with PBS for three times. Finally, the cells were incubated in the dark or irradiated at ambient temperature for 20 min. The light source consisted of a 300 W halogen lamp, a water tank for cooling, and a glass filter with a cut-on wavelength between 400 and 700 nm. The fluence rate was 23 mW cm^–2^. Illumination of 20 min led to a total fluence of 28 J cm^–2^. The fluorescence of DCF in these cells was imaged using confocal fluorescence microscopy. The DCF was excited at 488 nm, and the fluorescence was monitored at 500–580 nm.

### Flow Cytometric Analysis

Approximately 4 × 10^5^ HT29, A549, and HEK293 cells in DMEM or RPMI 1640 medium (2 mL) were seeded on a 6-well plate and incubated overnight at 37 °C in a humidified 5% CO_2_ atmosphere. After removal of the medium, the cells were treated with the conditions as described above for the confocal microscopic studies. After removing the medium and being rinsing with PBS for three times, the cells were harvested by 0.25% trypsin-ethylenediaminetetraacetic acid (0.4 mL). The activity of trypsin was quenched with the culture medium (0.5 mL), and the mixture was centrifuged at 1500 rpm for 3 min. The pellet was washed with PBS (1 mL) and then centrifuged. The cells were suspended in HBSS (1.0 mL) and then subject to flow cytometric analysis using a BD FACSVerse flow cytometer (Becton Dickinson) with 10^4^ cells counted in each sample. Cell fragments were excluded with a forward and side-scatter gating to ensure that all the detected signals were originated from the relatively intact cells. The signals from the BODIPY units were recorded in Chanel FITC. All experiments were performed in triplicate.

### Study of Photocytotoxicity

Approximately 1 × 10^4^ HT29, A549, or R-HepG2 cells or 3 × 10^4^ HEK293 cells per well in the culture medium were inoculated in 96-well plates and incubated overnight at 37 °C in a humidified 5% CO_2_ atmosphere. The cells were then incubated with **1**, **2**, **3**, **4**, or **11** at various concentrations for 8 h. For the light treatment groups, the cells were irradiated with light coming from the aforementioned light source (λ = 400–700 nm, 23 mW cm^–2^, 28 J cm^–2^) for 20 min. Cell viability was determined by means of a colorimetric MTT assay. After illumination, the cells were incubated at 37 °C in a humidified 5% CO_2_ atmosphere for 16 h. An MTT (Sigma) solution in PBS (3 mg mL^–1^, 50 μL) was added to each well followed by incubation for 4 h under the same environment. After that, 70 μL of DMSO was added to each well. Solutions in all wells were mixed until homogenous. Absorbance at 490 nm was measured using a plate reader (Tecan Spark 10M Microplate Reader). The average absorbance of the blank wells, which did not contain the cells, was subtracted from the readings of the other wells. The cell viability was then determined by the equation: % viability = [(∑*A_i_*/*A*_control_ × 100)]/*n*, where *A_i_* is the absorbance of the *i*th datum (*i* = 1, 2···*n*), *A*_control_ is the average absorbance of the control wells, in which the drug was absent, and *n* (= 4) is the number of data points. The percentages of cell viabilities in the cytotoxicity experiments were used as the quantitative expression of the effect of the drugs.

### HPLC Analysis for 1 and 2

Reverse-phase HPLC analysis was performed on an Apollo-C18 column (5 μm, 4.6 mm × 150 mm) at a flow rate of 1 mL min^–1^, using a Waters system equipped with a Waters 1525 binary pump and a Waters 2998 photodiode array detector. The solvents used for the analysis were of HPLC grade. The conditions were set as follows: solvent A = 0.1% trifluoroacetic acid (TFA) in acetonitrile and solvent B = 0.1% TFA in deionized water. The gradient was 100% B in the first 5 min, changed to 100% A in 30 min, maintained under this condition for 10 min, changed back to 100% B in 5 min, and then kept at this condition for 10 min. The purity was found to be >95% for both metallacycles **1** and **2**.

## Supporting Information Available

Comparison of the singlet oxygen generation efficiency of **1**–**4** in water, concentration-dependent absorption spectra of **3** and **4** in 1% DMSO in water, HPLC chromatograms of **1** and **2** in RMPI 1640 medium over 24 h, competition assay for **1** using free glucose, cellular uptake of **2**, and NMR and MS spectra of all the new compounds (PDF)
Molecular formula strings (CSV)

## Abbreviations Used

ABDA: 9,10-anthrancenediyl-bis(methylene)dimalonic acid
BODIPY: boron dipyrromethene
CI: combination index
DCF: dichlorofluorescein
DLS: dynamic light scattering
DMEM: Dulbecco’s modified Eagle medium
DMSO: dimethylsulfoxide
DOSY: diffusion-ordered spectroscopy
DPBF: 1,3-diphenylisobenzofuran
ESI-MS: electrospray ionization mass spectrometry
FBS: fetal bovine serum
GLUT: glucose transporter
H_2_DCFDA: 2′,7′-dichlorodihydrofluorescein diacetate
HBSS: Hank’s Balanced Salt Solution; HPLC, high-performance liquid chromatography
IC_50_: half-maximal inhibitory concentrations
ICI: iodine monochloride
MALDI-TOF: matrix-assisted laser desorption/ionization time-of-flight
MTT: 3-(4,5-dimethylthiazol-2-yl)-2,5-diphenyltetrazolium bromide
PBS: phosphate-buffered saline
PDI: polydispersity index
PDT: photodynamic therapy
PS: photosensitizers
ROS: reactive oxygen species
RPMI: Roswell Park Memorial Institute
SD: standard deviation
SEM: standard error of the mean
TEG: tetraethylene glycol
TEM: transmission electron microscopy
TLC: thin layer chromatography
UV−vis: ultraviolet and visible
Φ_Δ_: singlet oxygen quantum yield
