# Supplementary material for: Glycosylated BODIPY- Incorporated Pt(II) Metallacycles for Targeted and Synergistic Chemo-Photodynamic Therapy
Source: J Med Chem. 2023 Feb 21;66(5):3448–59. doi: 10.1021/acs.jmedchem.2c01940 (PMC10009748; doi:10.1021/acs.jmedchem.2c01940)
Supplement: Supplementary file 1 — jm2c01940_si_001.pdf [file jm2c01940_si_001.pdf]

Supporting Information

Glycosylated BODIPY-Incorporated Pt(II) Metallacycles for Targeted and Synergistic Chemo-Photodynamic Therapy

Gonzalo Durán-Sampedro,[†,§,#] Evelyn Y. Xue,[‡,#] Marta Moreno-Simoni,[†] Celia Paramio,[†] Tomás Torres,[†,§,¶,*] Dennis K. P. Ng,[‡,*] and Gema de la Torre[†,§,*]

[†] *Department of Organic Chemistry, Universidad Autónoma de Madrid, Campus de Cantoblanco, Madrid 28049, Spain*

[§] *Institute for Advanced Research in Chemical Sciences (IAdChem), Universidad Autónoma de Madrid, Campus de Cantoblanco, Madrid 28049, Spain*

[‡] *Department of Chemistry, The Chinese University of Hong Kong, Shatin, N.T., Hong Kong, China*

¶ *IMDEA Nanociencia, C/Faraday 9, Cantoblanco, Madrid 28049, Spain*

[#] *These authors contributed equally to this work.*

Corresponding author: Gema de la Torre. Email: gema.delatorre@uam.es

Table of Contents

1. **Supplementary figures** S2

2. **NMR spectra** S7

3. **Mass spectra** S16

4. HPLC chromatograms...S20

1. Supplementary figures

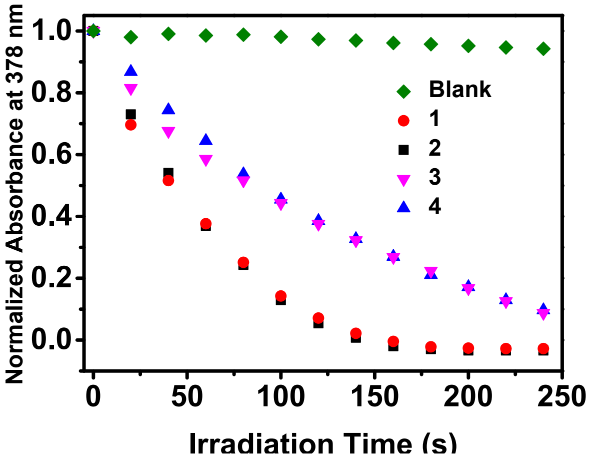

Figure S1. Rates of decay of ABDA (initial concentration = 50 μM), as monitored spectroscopically at 378 nm, in the absence and presence of **1** (4 μM), **2** (4 μM), **3** (12 μM), or **4** (12 μM) in water upon irradiation (λ = 400−700 nm).

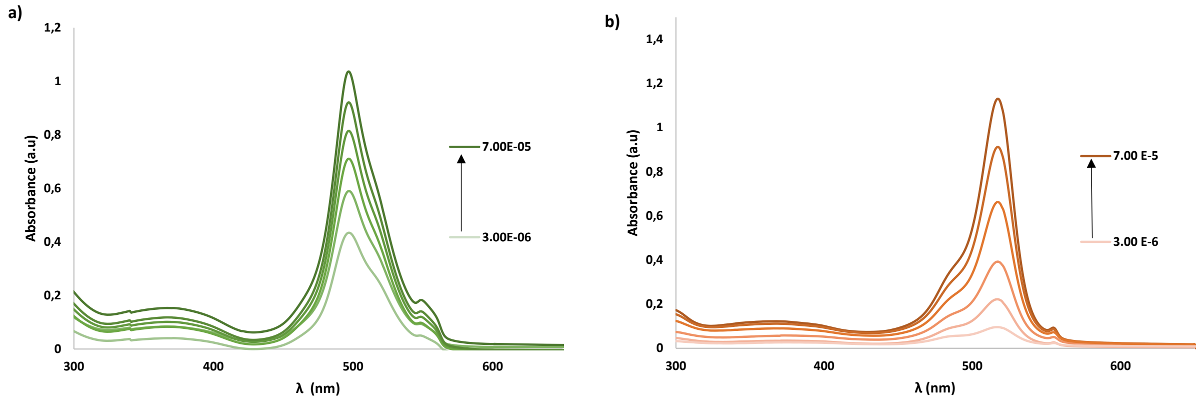

Figure S2. Concentration-dependent absorption spectra of (a) **3** and (b) **4** in 1% DMSO in water.

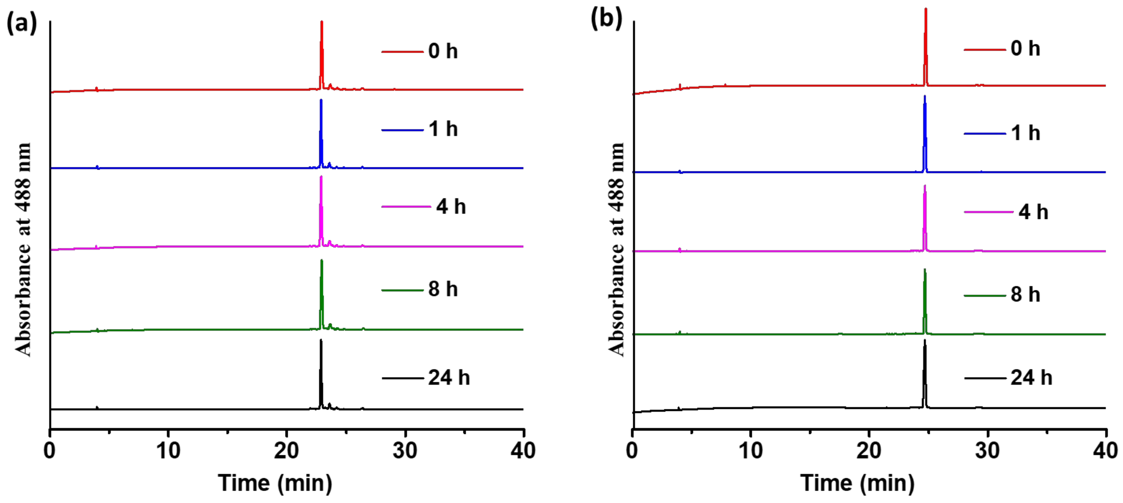

Figure S3. HPLC chromatograms of (a) **1** and (b) **2** in RPMI 1640 medium at 37 ^{o}C recorded at different time points over a period of 24 h.

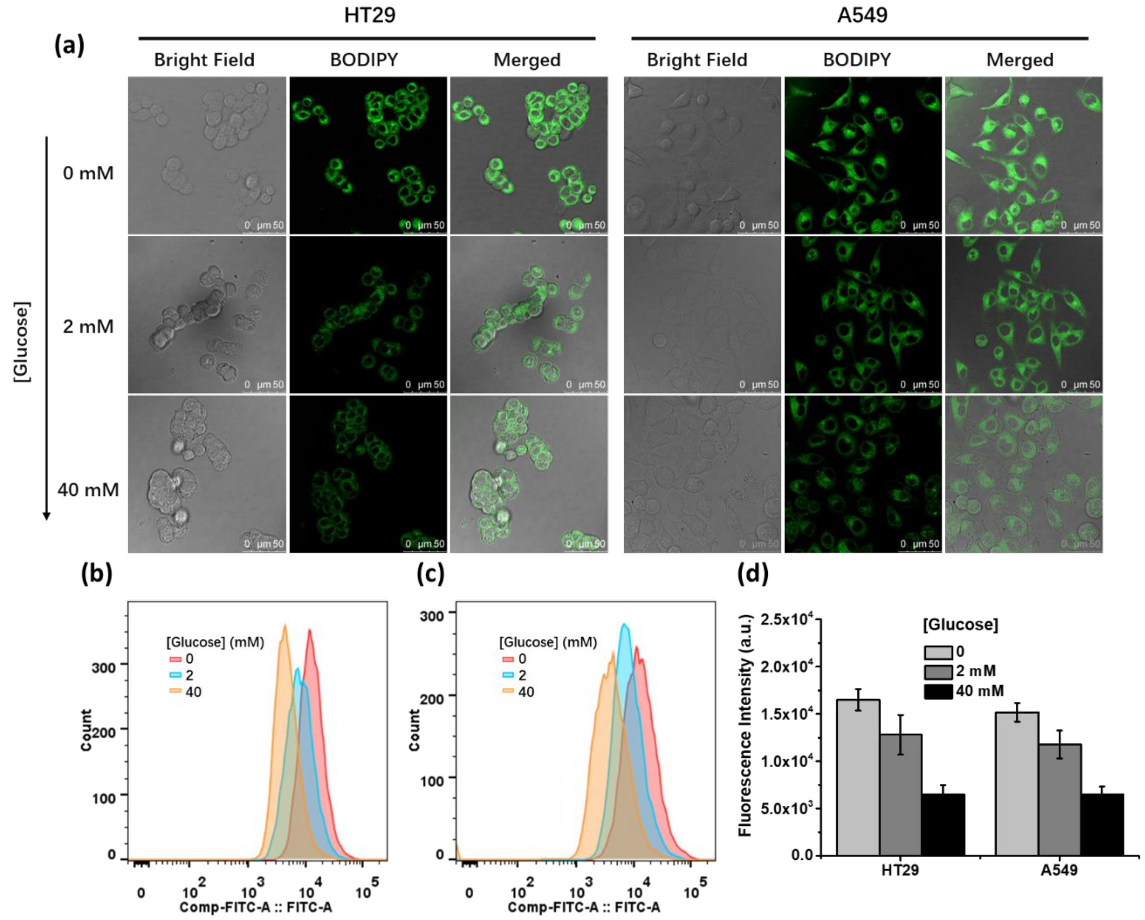

Figure S4. (a) Bright field, fluorescence, and the merged confocal images of HT29 and A549 cells after incubation with **1** (4 µM) with or without the presence of free glucose (2 and 40 mM) for 1 h. Fluorescence intensity profiles of (b) HT29 and (c) A549 cells being treated under these conditions determined by flow cytometry. Figure (d) shows the corresponding quantified intracellular fluorescence intensities. Data are expressed as the mean ± SD of three independent experiments.

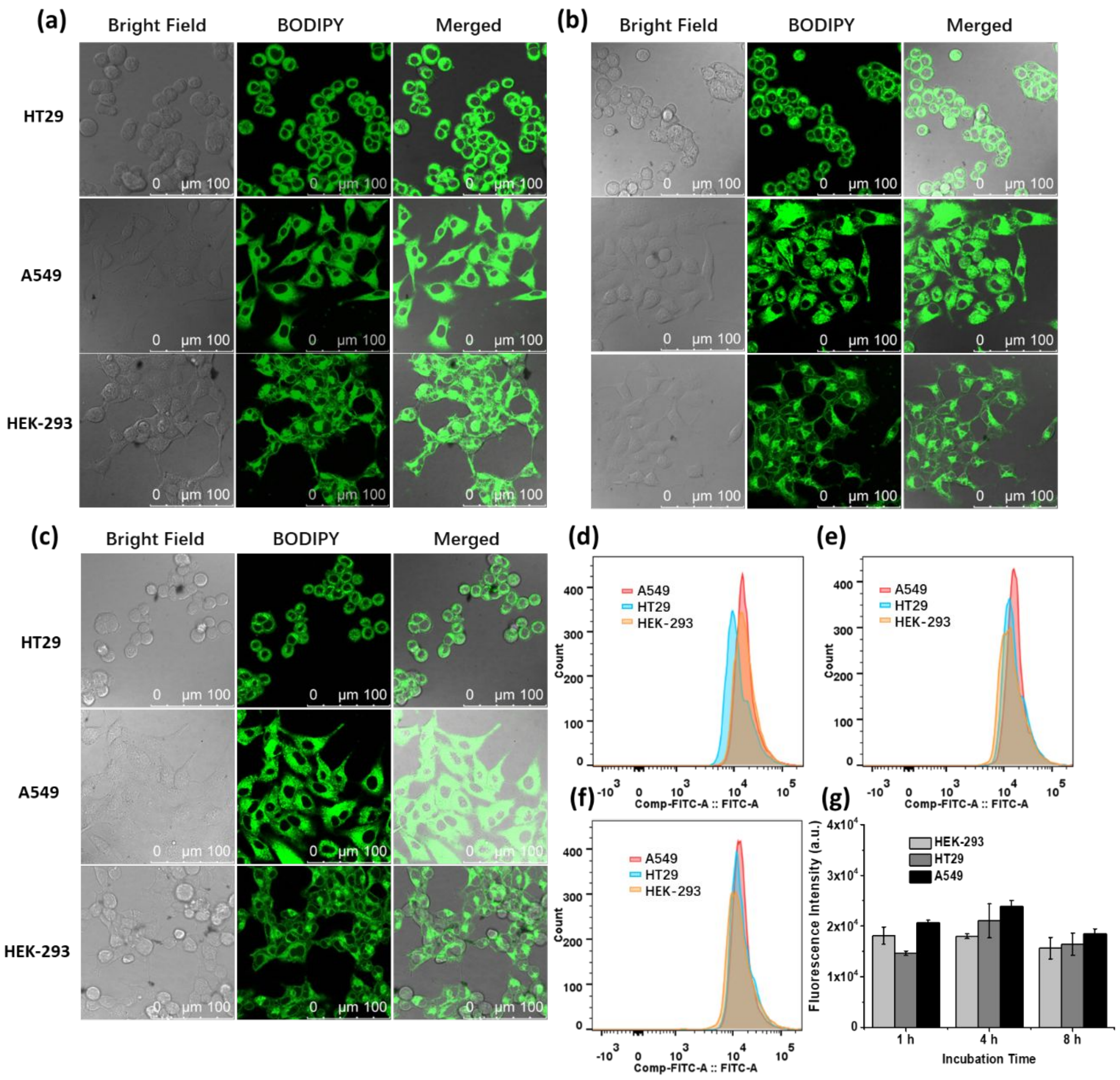

Figure S5. Bright field, fluorescence, and the merged confocal images of HT29, A549, and HEK-293 cells after incubation with **2** (4 μM) for (a) 1 h, (b) 4 h, and (c) 8 h, respectively. Figures (d)-(f) show the fluorescence intensity profiles of the cells being treated under these conditions, respectively, determined by flow cytometry. Figure (g) shows the corresponding quantified intracellular fluorescence intensities. Data are expressed as the mean ± SD of three independent experiments.

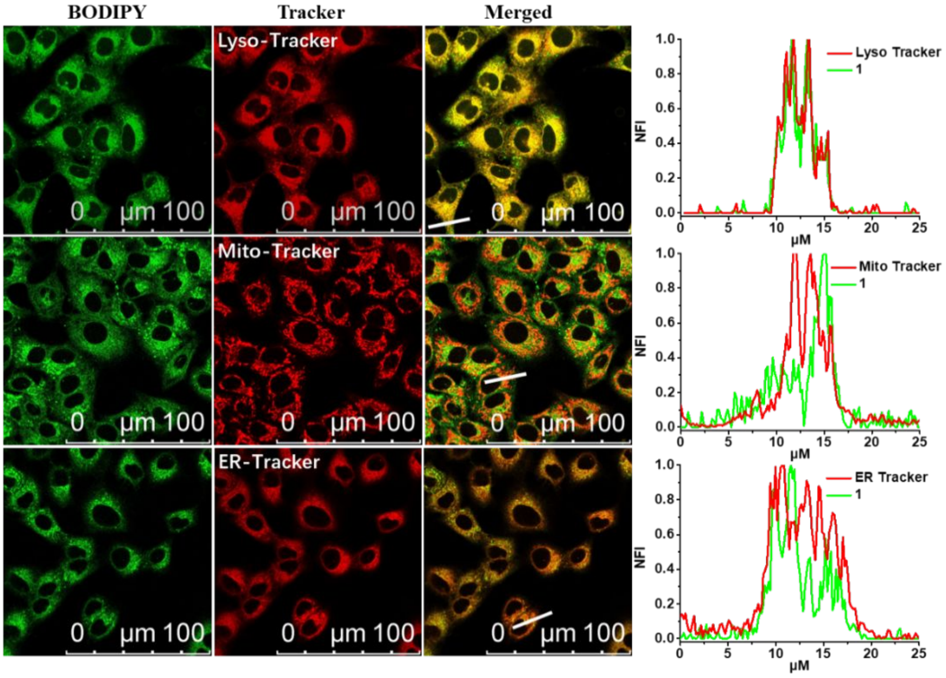

Figure S6. Confocal images of A549 cells after incubation with **1** (4 μM) for 1 h, followed by incubation with LysoTracker Deep Red (0.1 μM) for 30 min, MitoTracker Red CMXRos (0.1 μM) for 20 min, or ER-Tracker Red (1 μM) for 20 min. The rightmost figures show the normalized fluorescence intensity (NFI) profiles of **1** and the trackers traced along the white line in the corresponding merged image.

2. NMR spectra

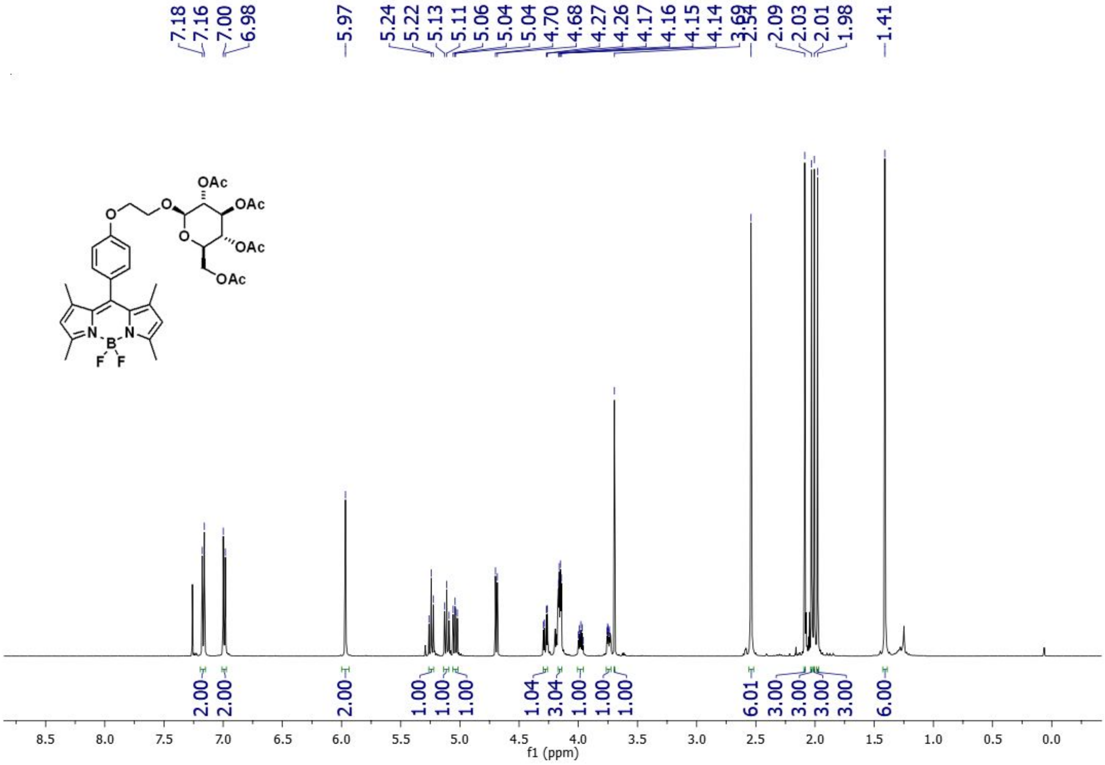

Figure S7. ^{1}H-NMR spectrum (300 MHz, $CDCl_3$) of **8a**

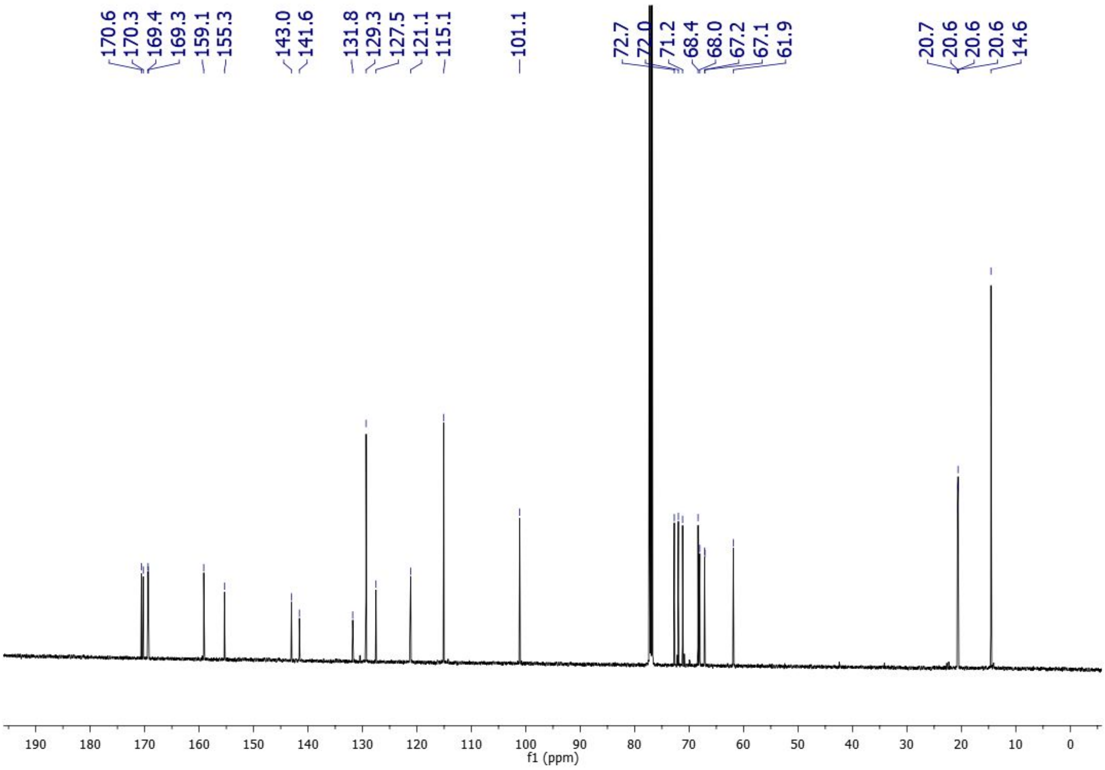

Figure S8. ^{13}C-NMR spectrum (126 MHz, $CDCl_3$) of **8a**

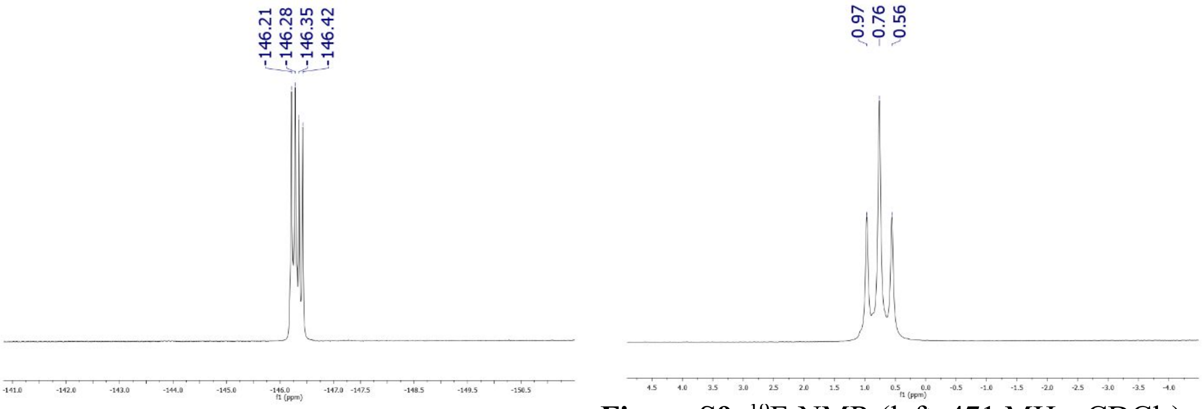

Figure S9. ^{19}F-NMR (left, 471 MHz, $CDCl_3$) and ^{11}B-NMR (right, 160 MHz, $CDCl_3$) spectra of **8a**

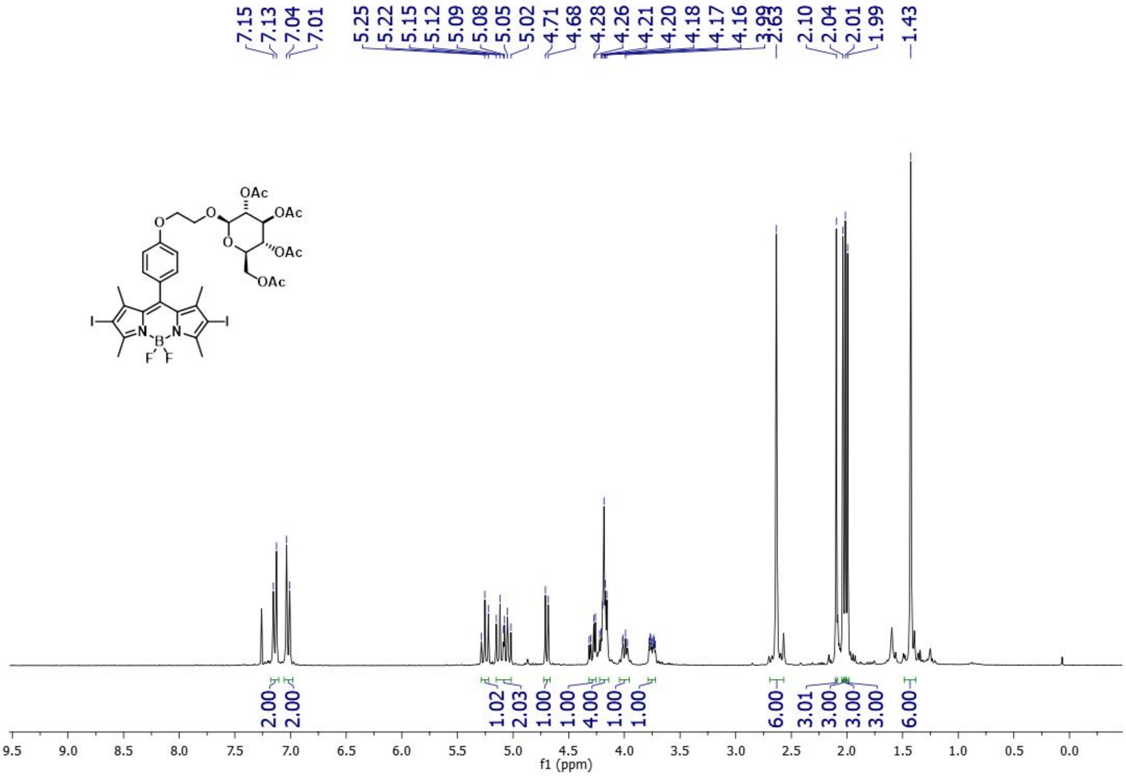

Figure S10. ^{1}H-NMR spectrum (300 MHz, $CDCl_3$) of **9a**

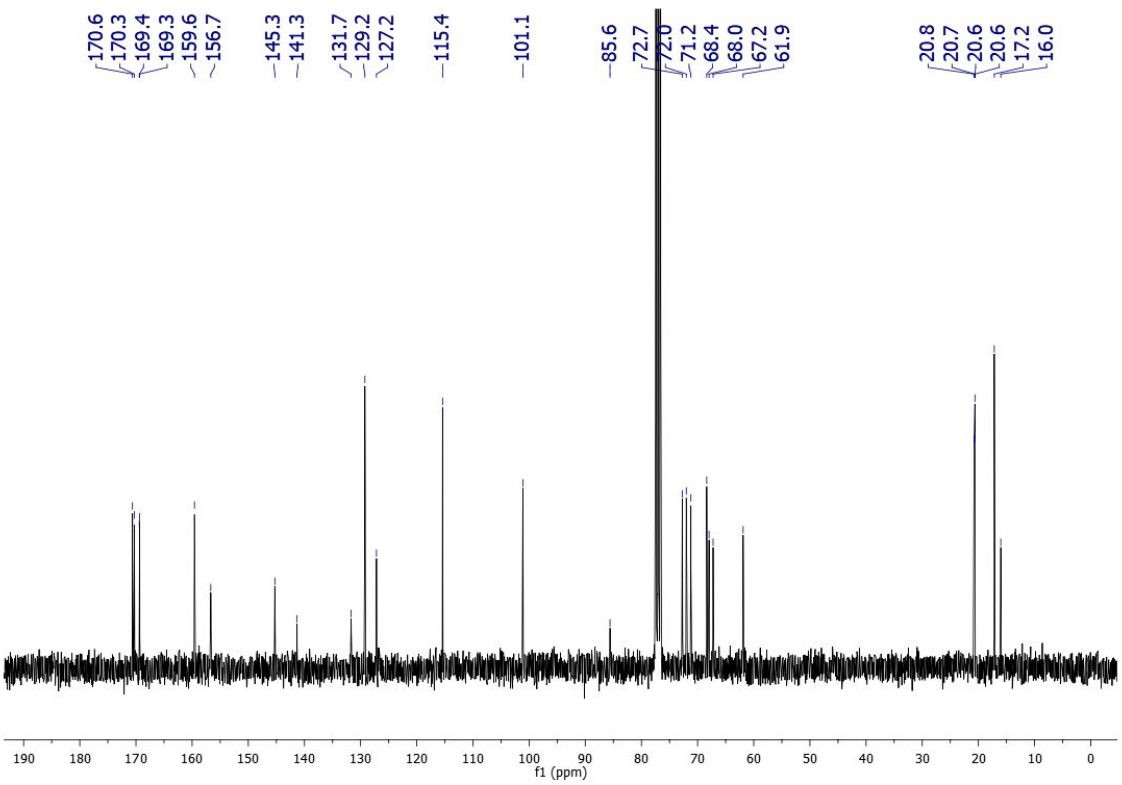

Figure S11. ^{13}C-NMR spectrum (75 MHz, $CDCl_3$) of **9a**

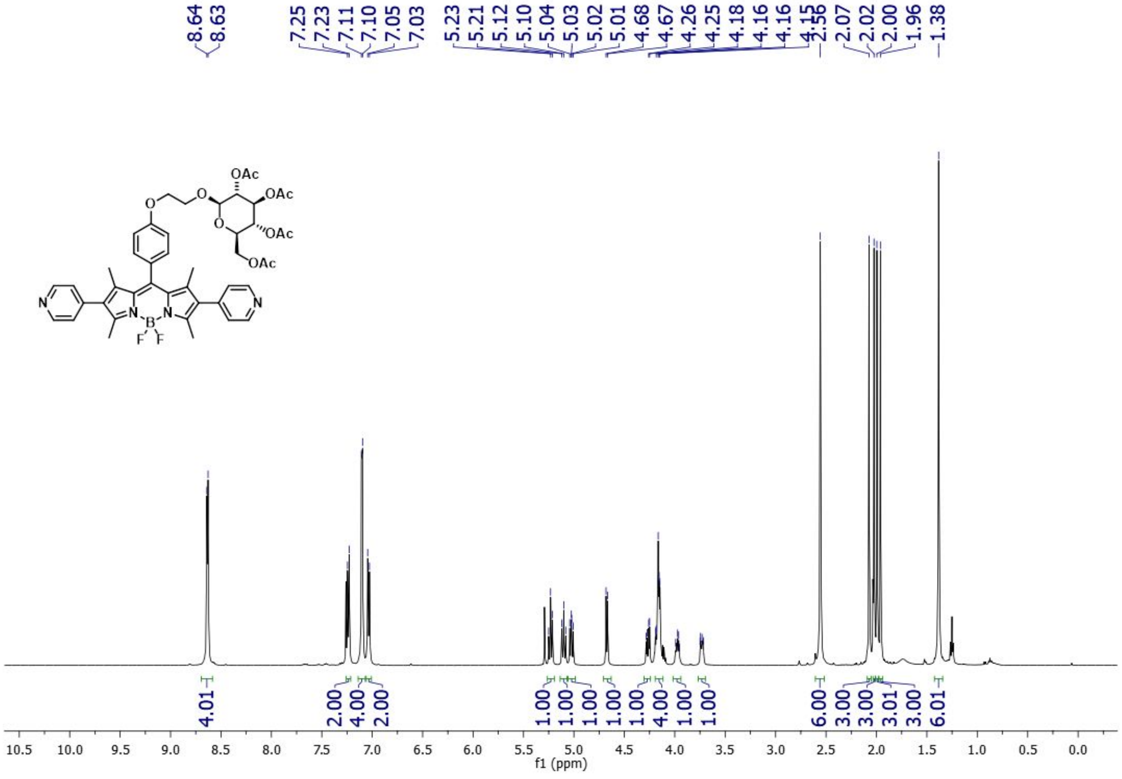

Figure S12. ^{1}H-NMR spectrum (500 MHz, $CDCl_3$) of **10**

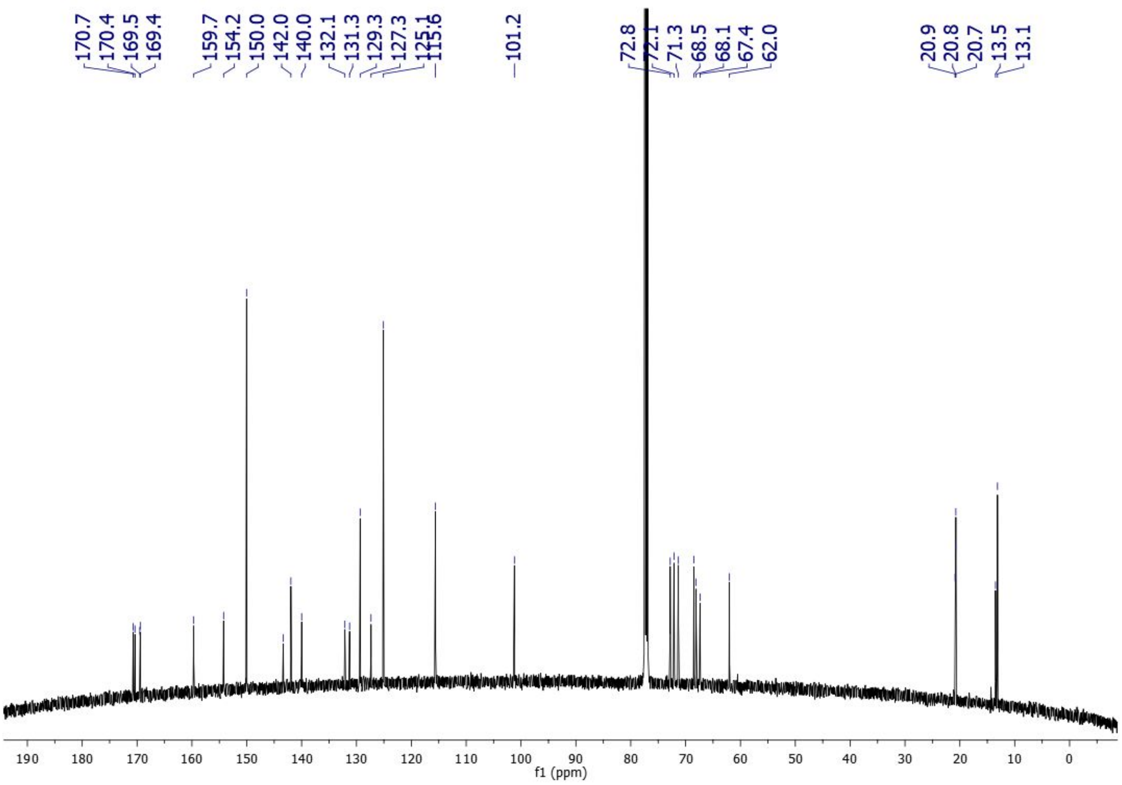

Figure S13. ^{13}C-NMR spectrum (126 MHz, $CDCl_3$) of **10**

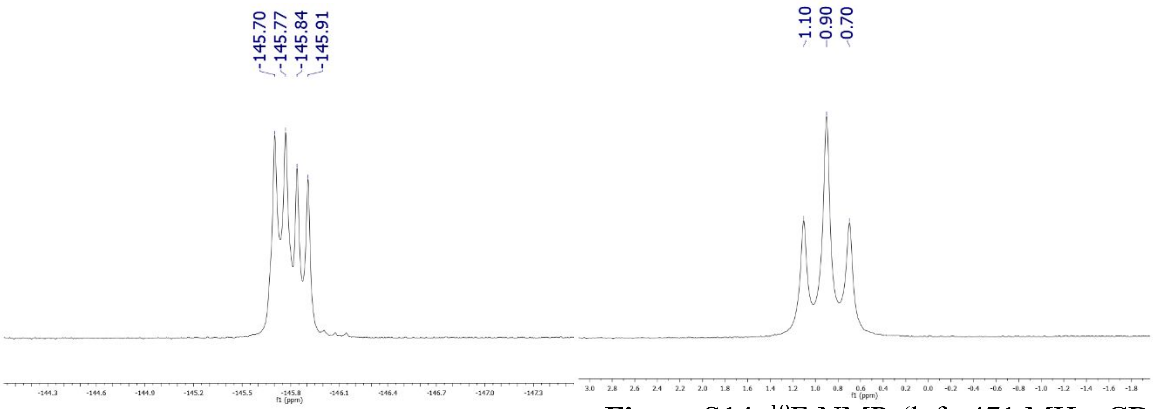

Figure S14. ^{19}F-NMR (left, 471 MHz, $CDCl_3$) and ^{11}B-NMR (right, 160 MHz, $CDCl_3$) spectra of **10**

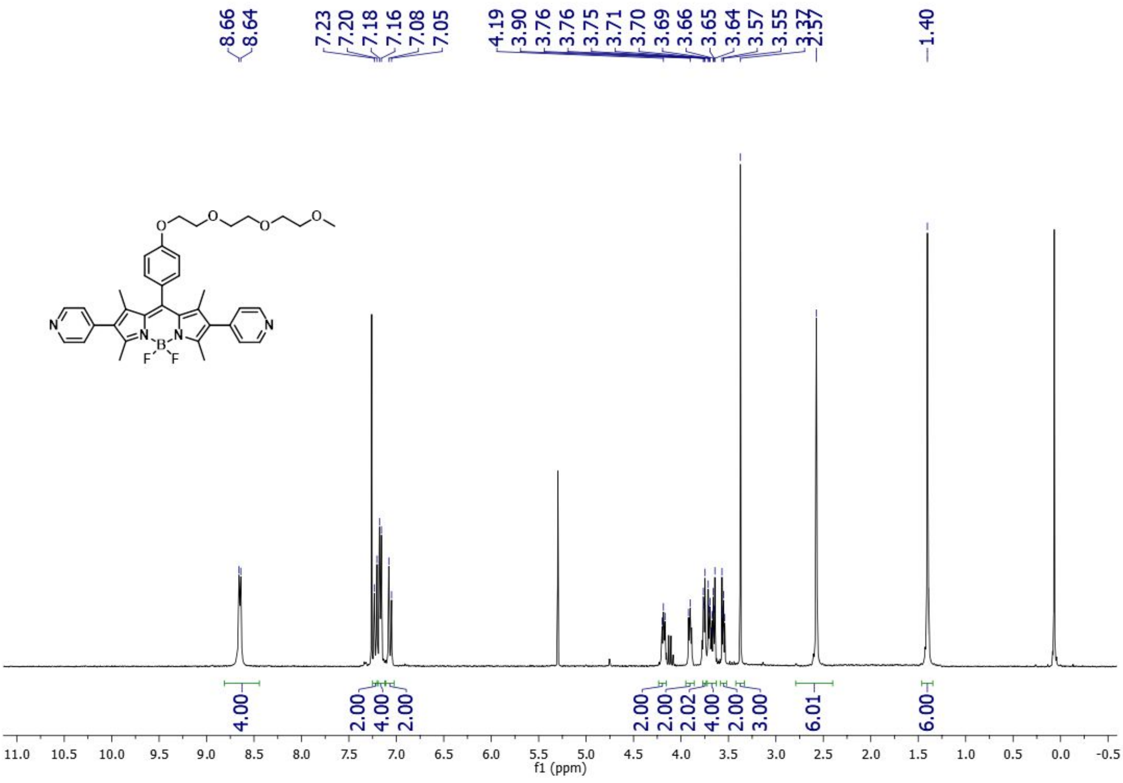

Figure S15. ^{1}H-NMR spectrum (300 MHz, $CDCl_3$) of **4**

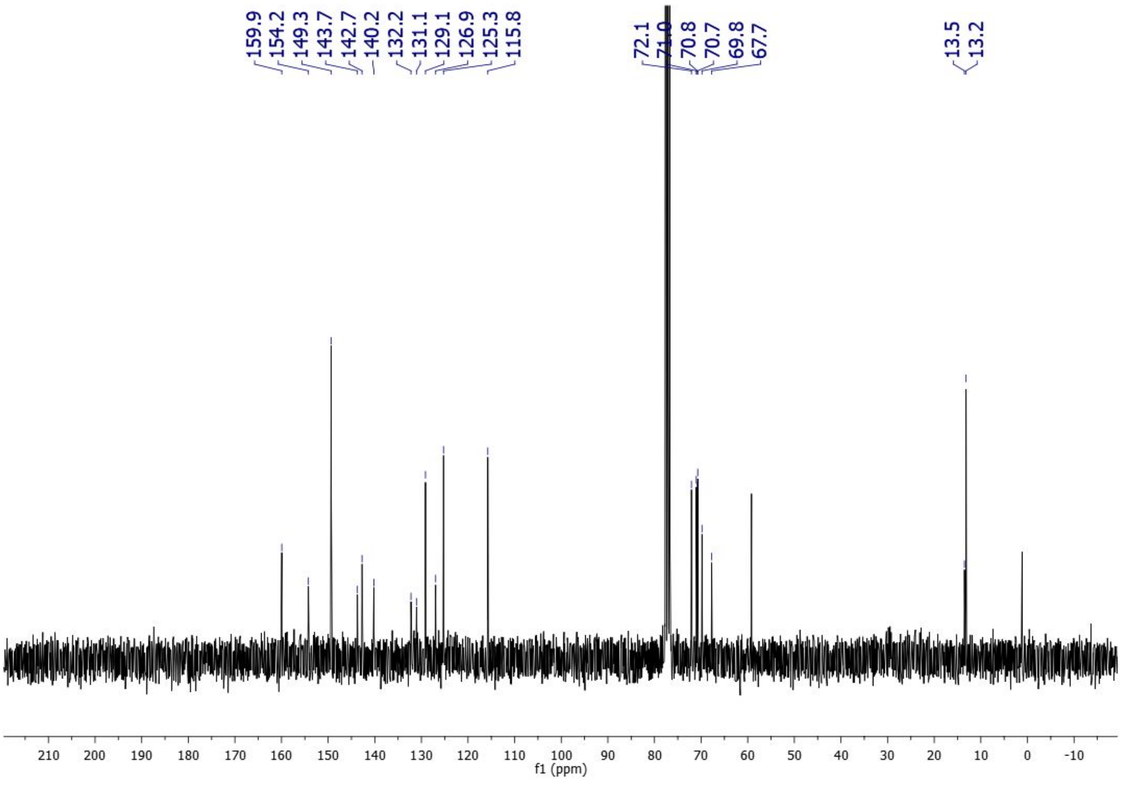

Figure S16. ^{13}C-NMR spectrum (75 MHz, $CDCl_3$) of **4**

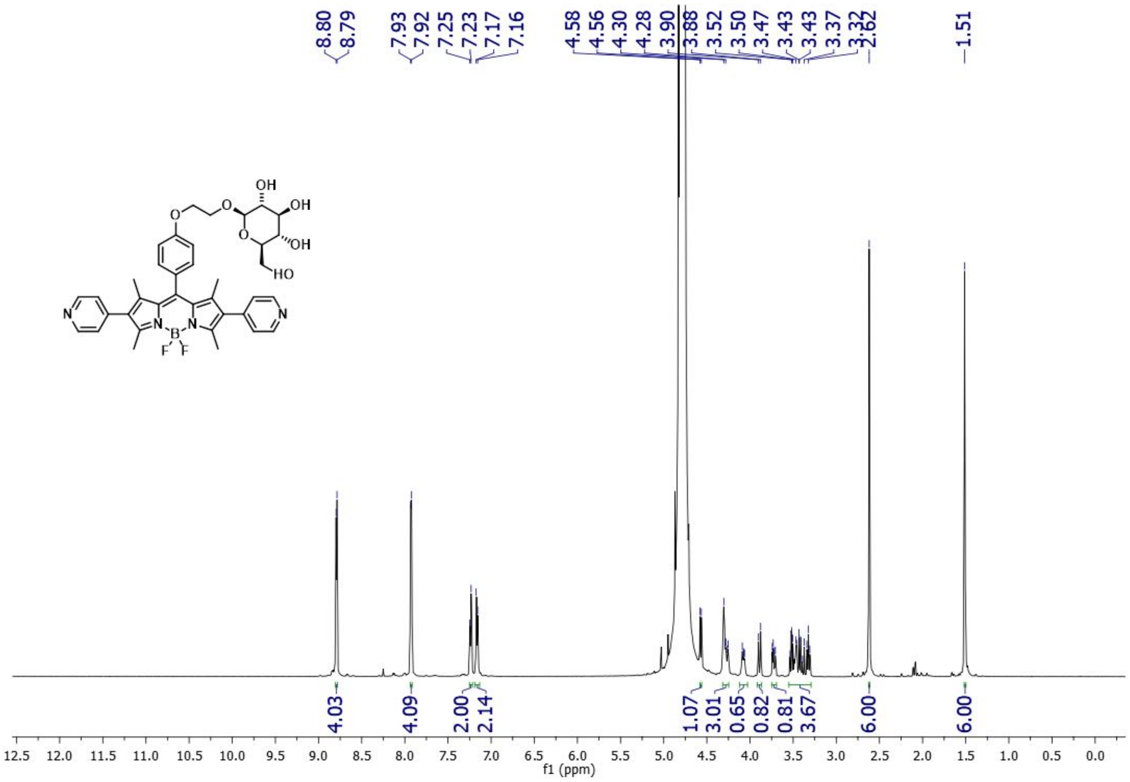

Figure S17. ^{1}H-NMR spectrum (300 MHz, D_2O) of **3**

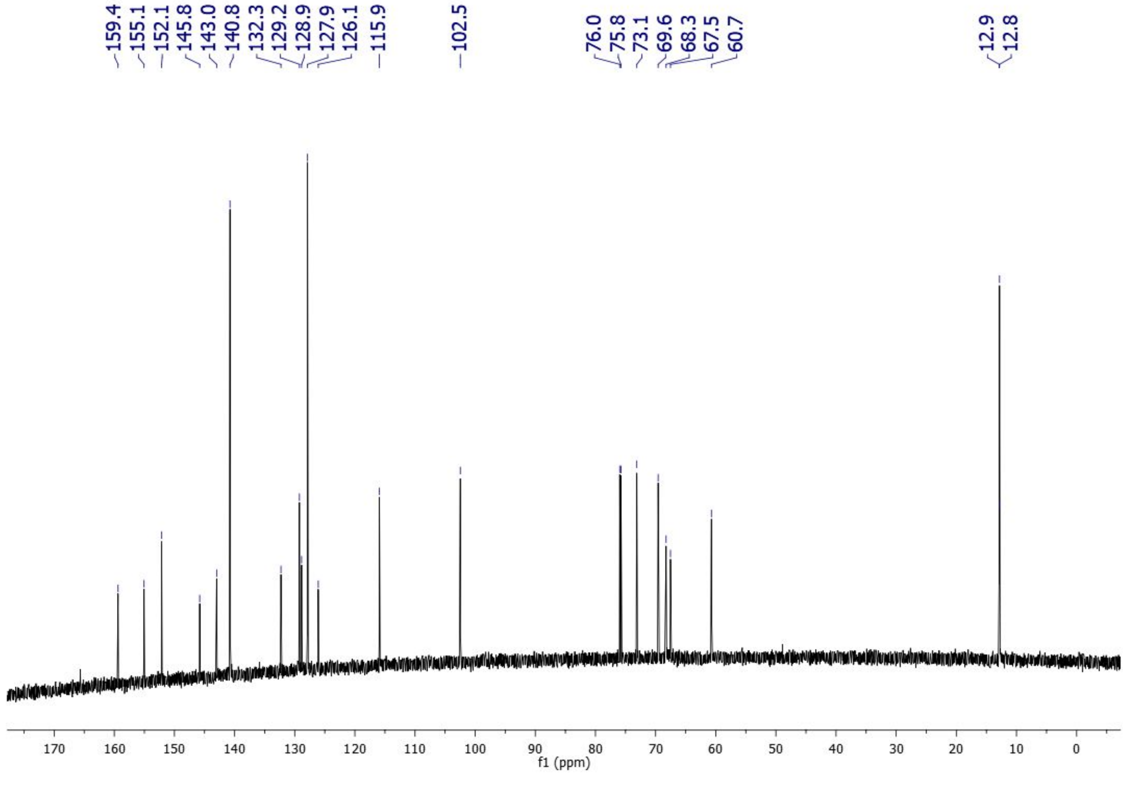

Figure S18. ^{13}C-NMR spectrum (126 MHz, D_2O) of **3**

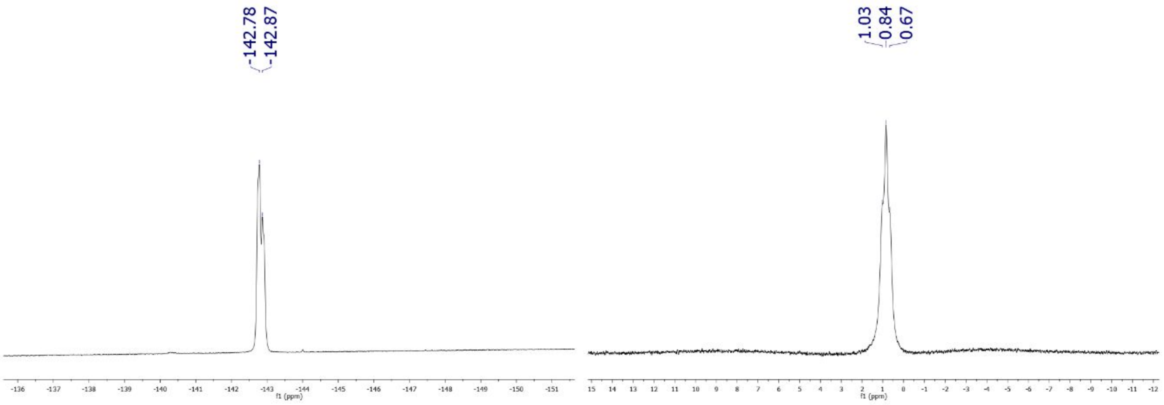

Figure S19. ^{19}F-NMR (left, 126 MHz, D_2O) and ^{11}B-NMR (right, 160 MHz, D_2O) spectra of **3**

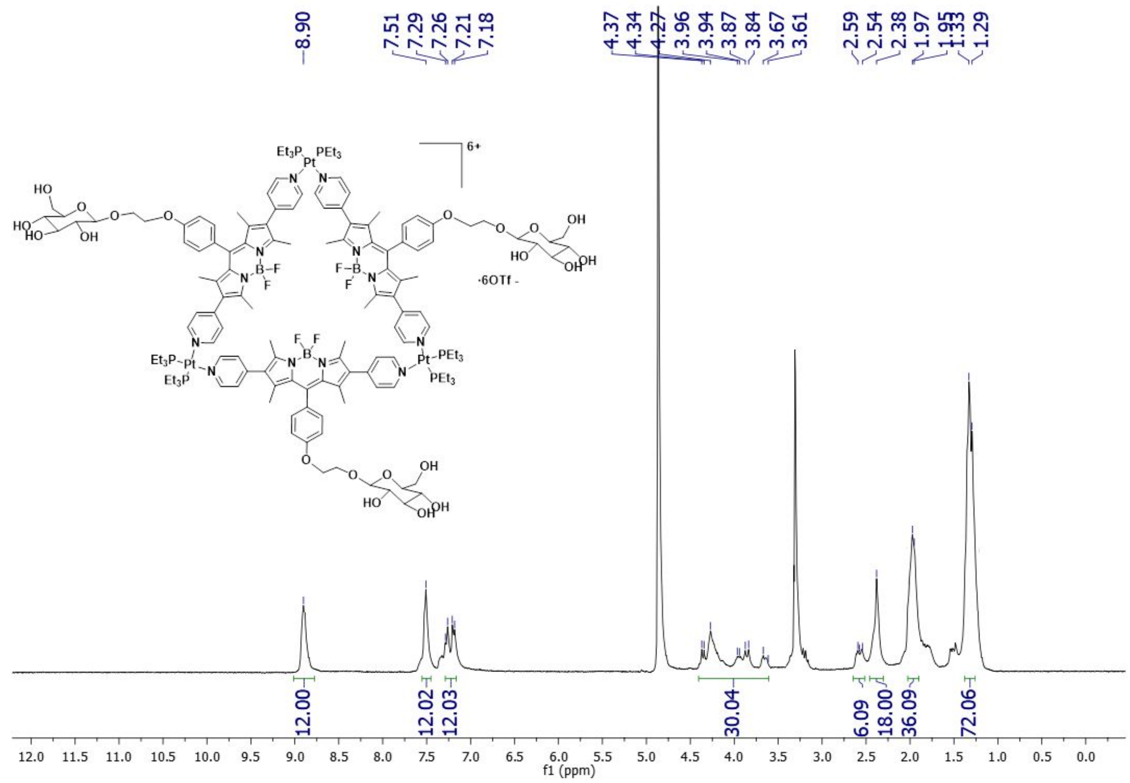

Figure S20. ^{1}H-NMR spectrum (300 MHz, CD_3OD) of **1**

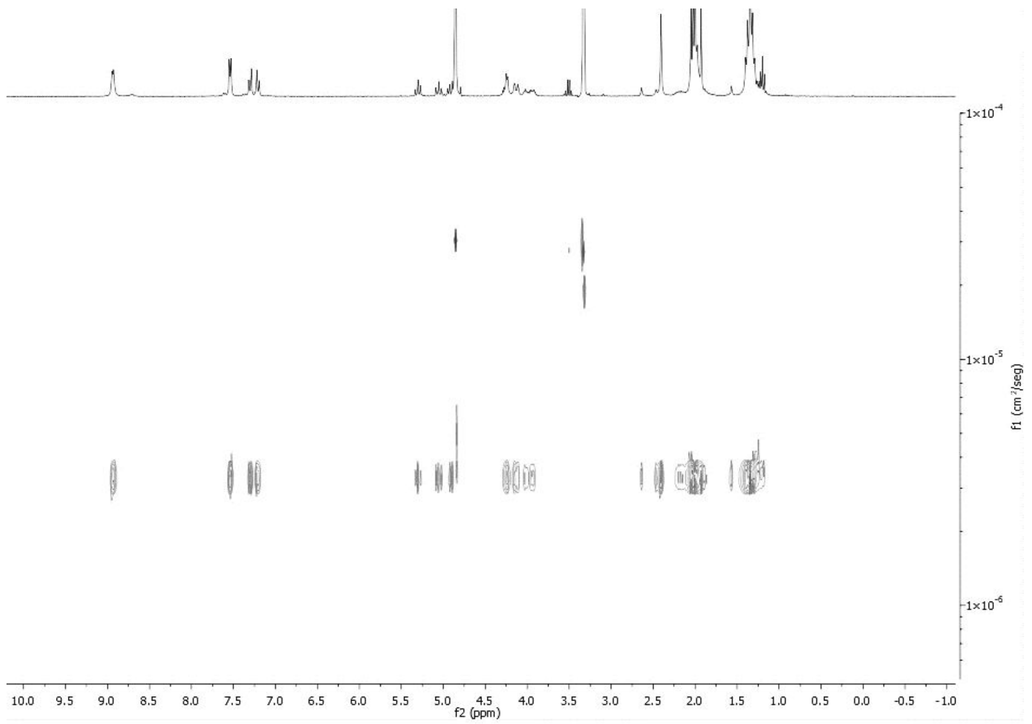

Figure S21. DOSY NMR spectrum (300 MHz, CD_3OD) of **1**

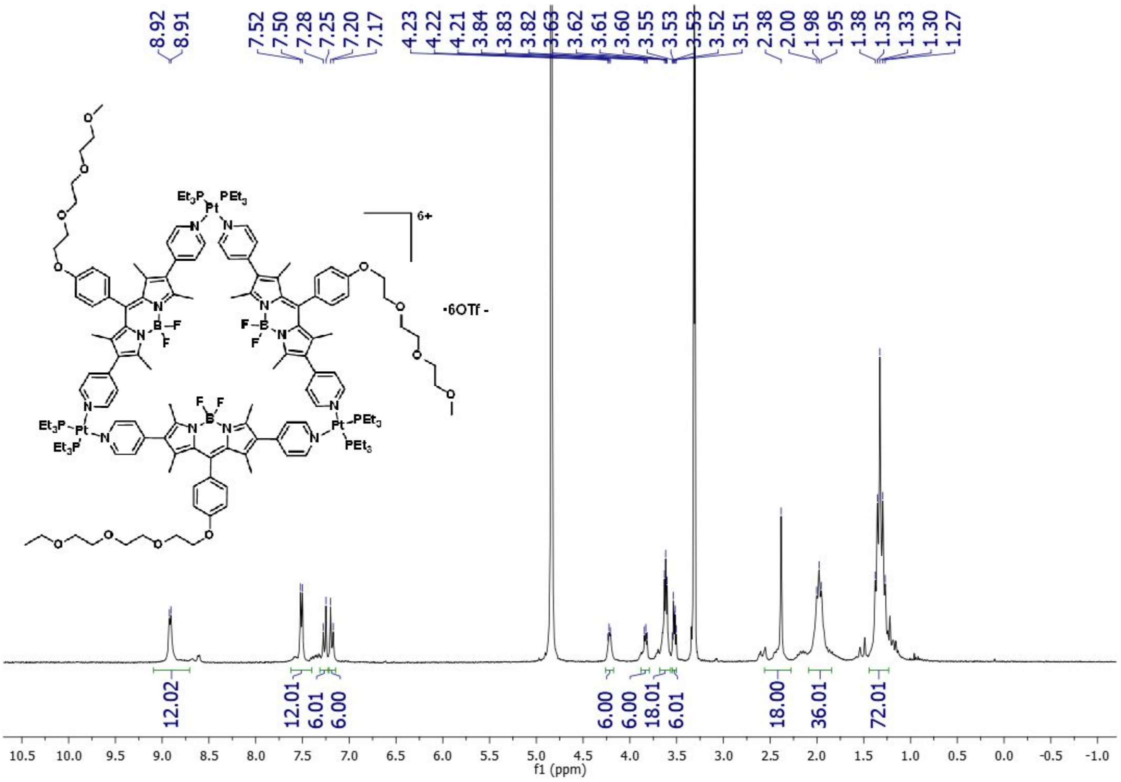

Figure S22. ^{1}H-NMR spectrum (300 MHz, CD_3OD) of **2**

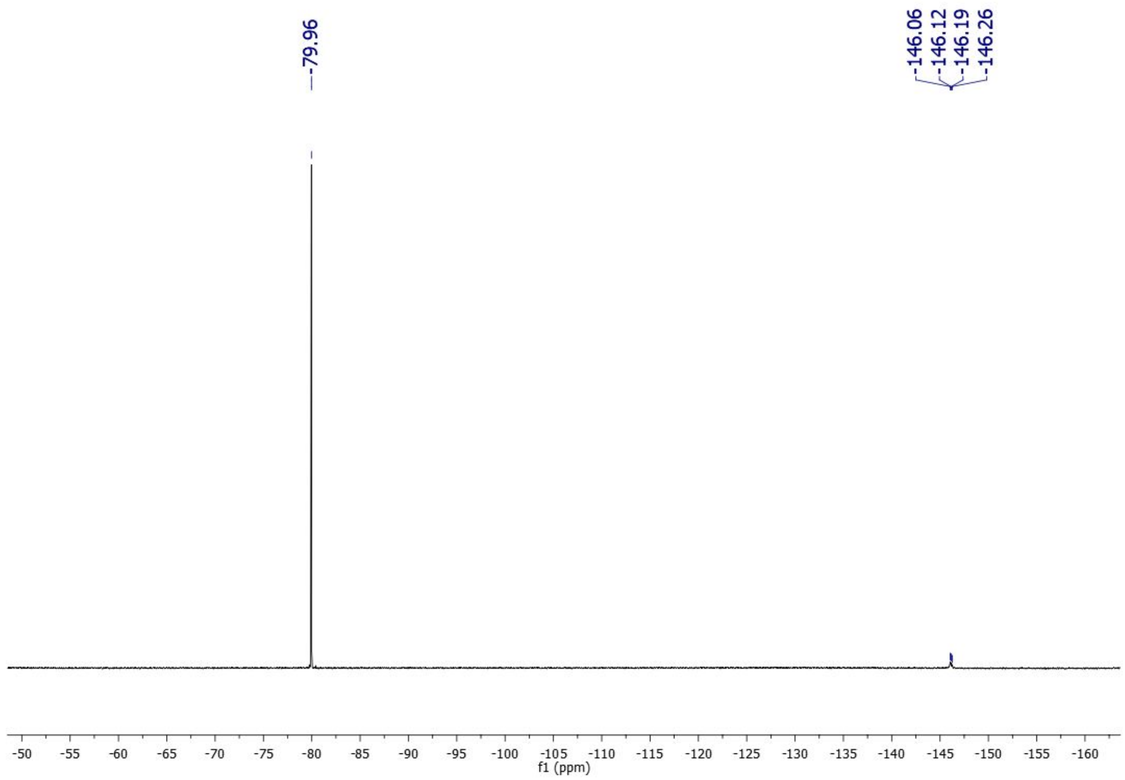

Figure S23. ^{19}F-NMR spectrum (471 MHz, CD_3OD) of **2**

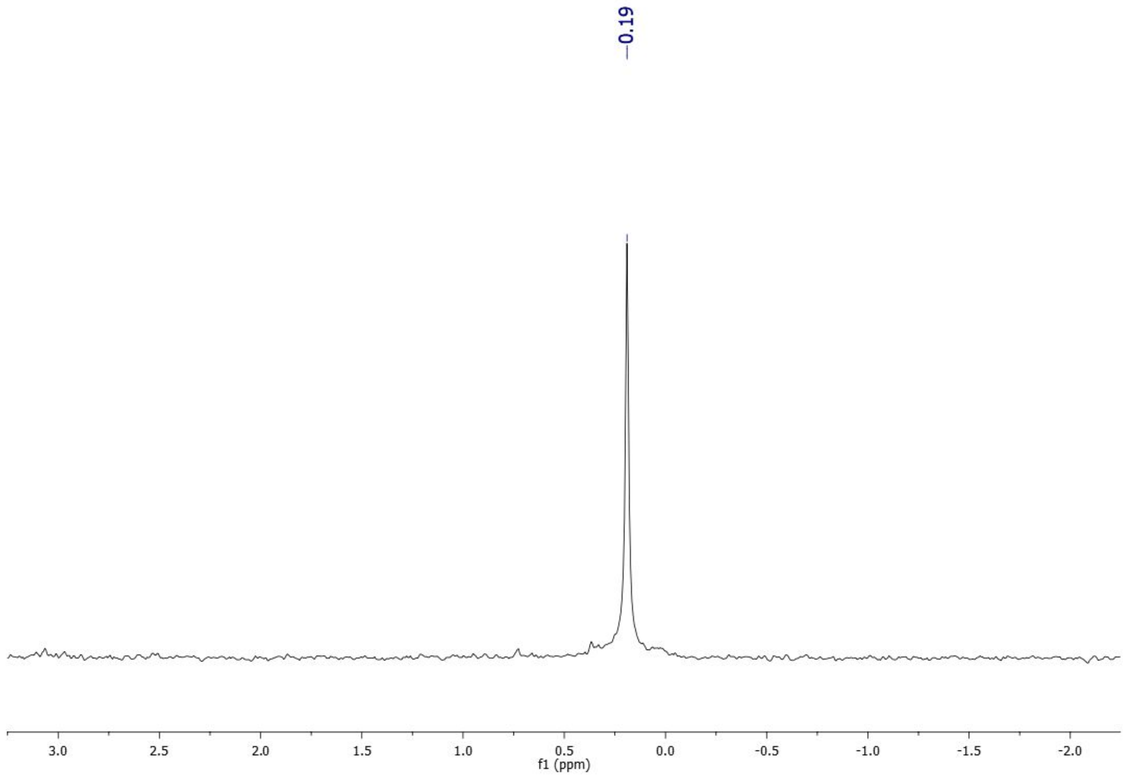

Figure S24. ^{31}P-NMR spectrum (202 MHz, acetone-d_6) of **2**

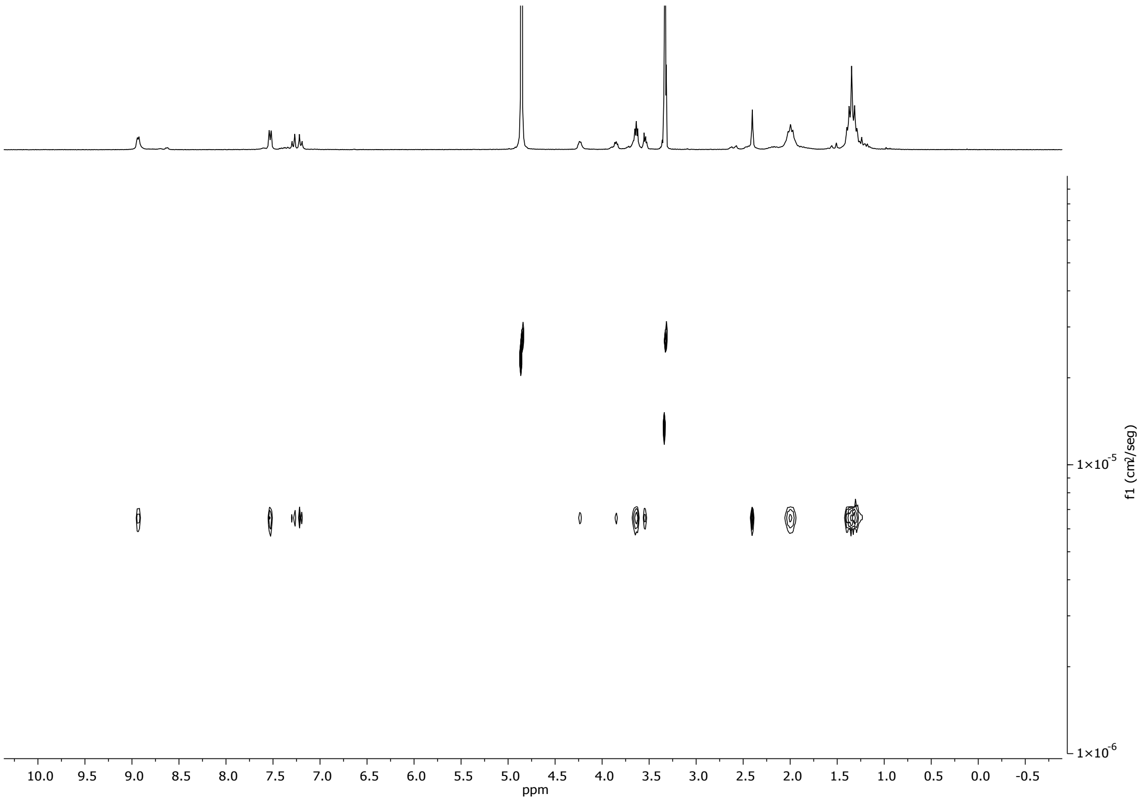

Figure S25. DOSY NMR spectrum (300 MHz, CD_3OD) of **2**

3. Mass spectra

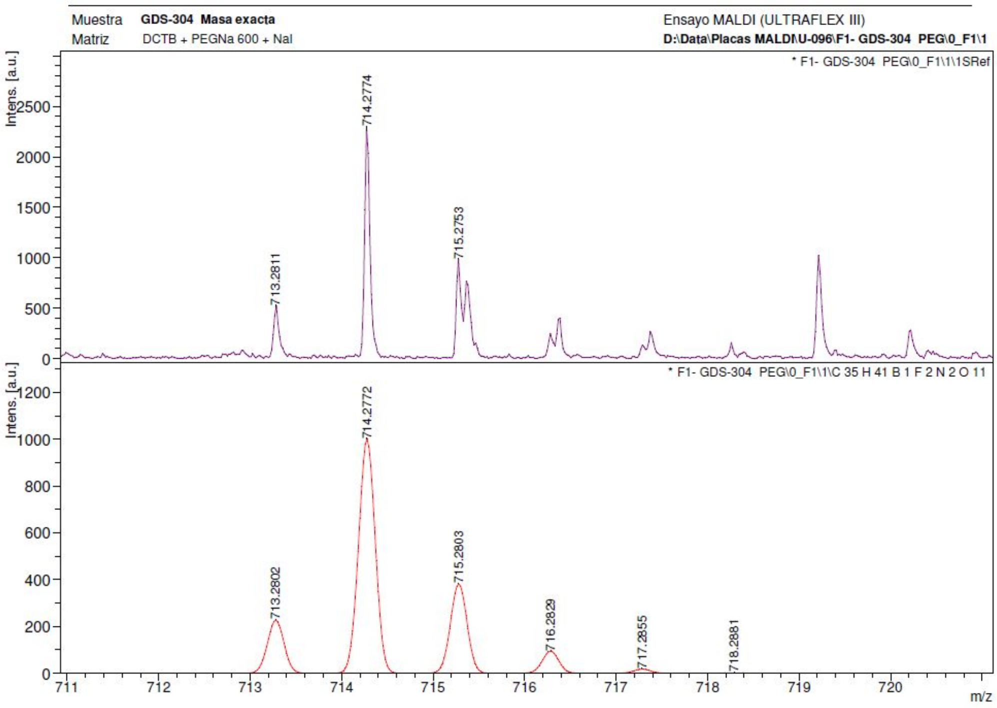

Figure S26. Experimental (top) and theoretical (bottom) isotopic patterns of the molecular ion of **8a** shown in the MALDI-TOF mass spectrum.

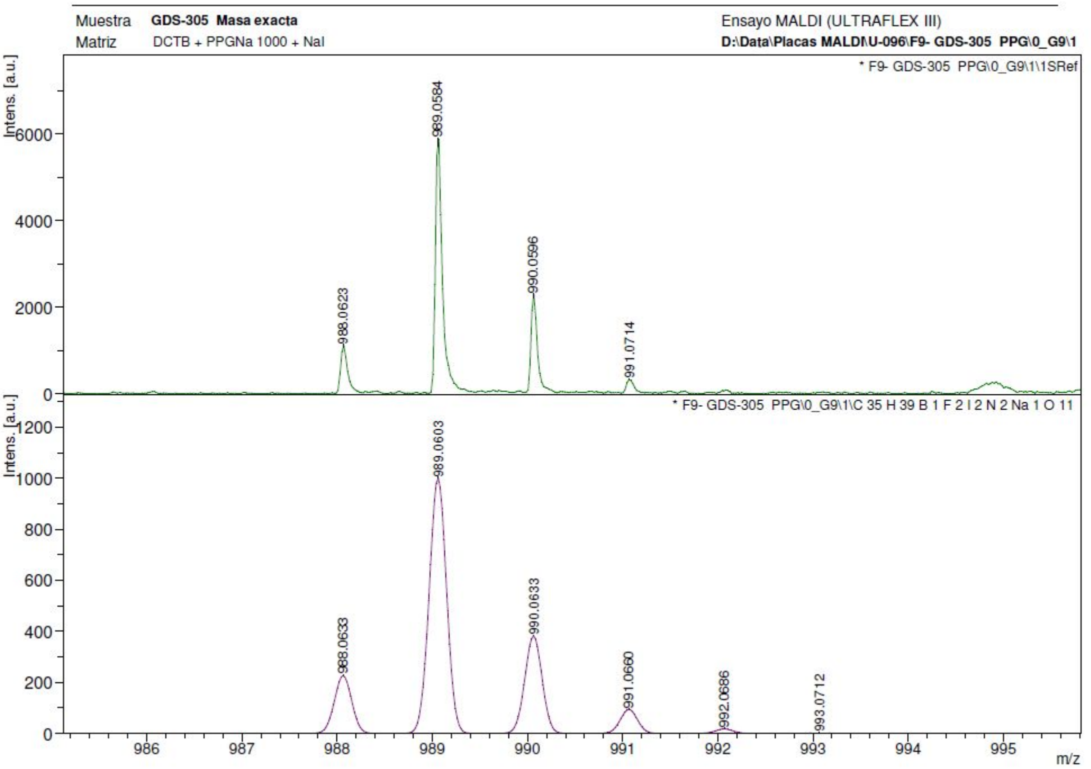

Figure S27. Experimental (top) and theoretical (bottom) isotopic patterns of the molecular ion of **9a** shown in the MALDI-TOF mass spectrum.

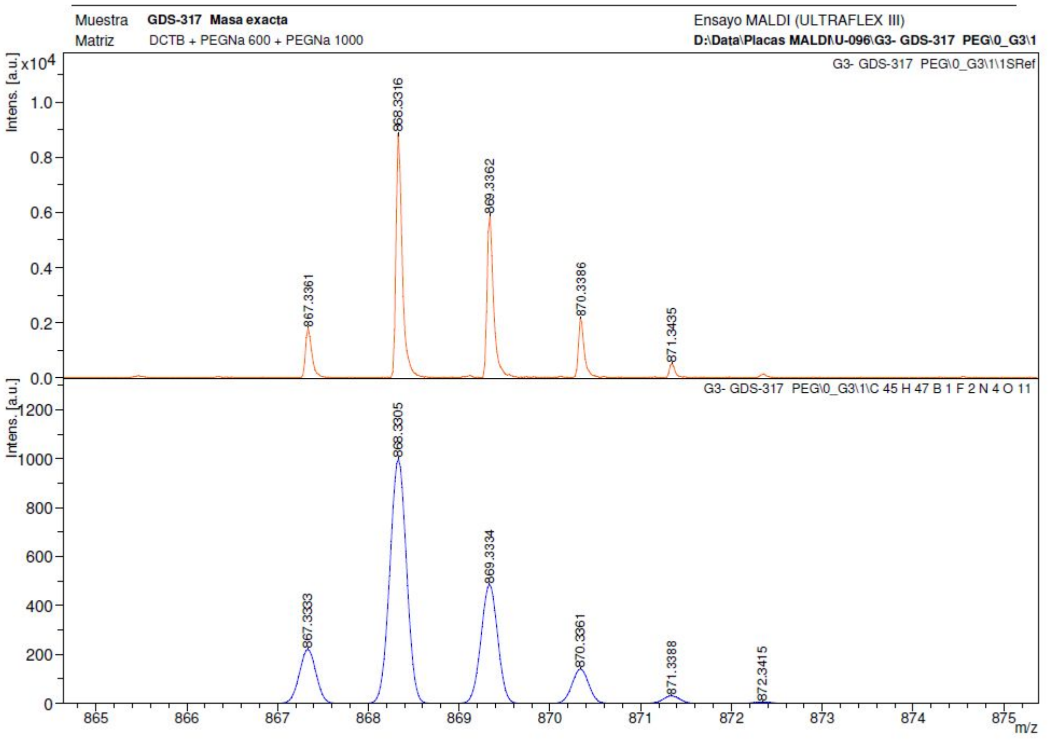

Figure S28. Experimental (top) and theoretical (bottom) isotopic patterns of the molecular ion of **10** shown in the MALDI-TOF mass spectrum.

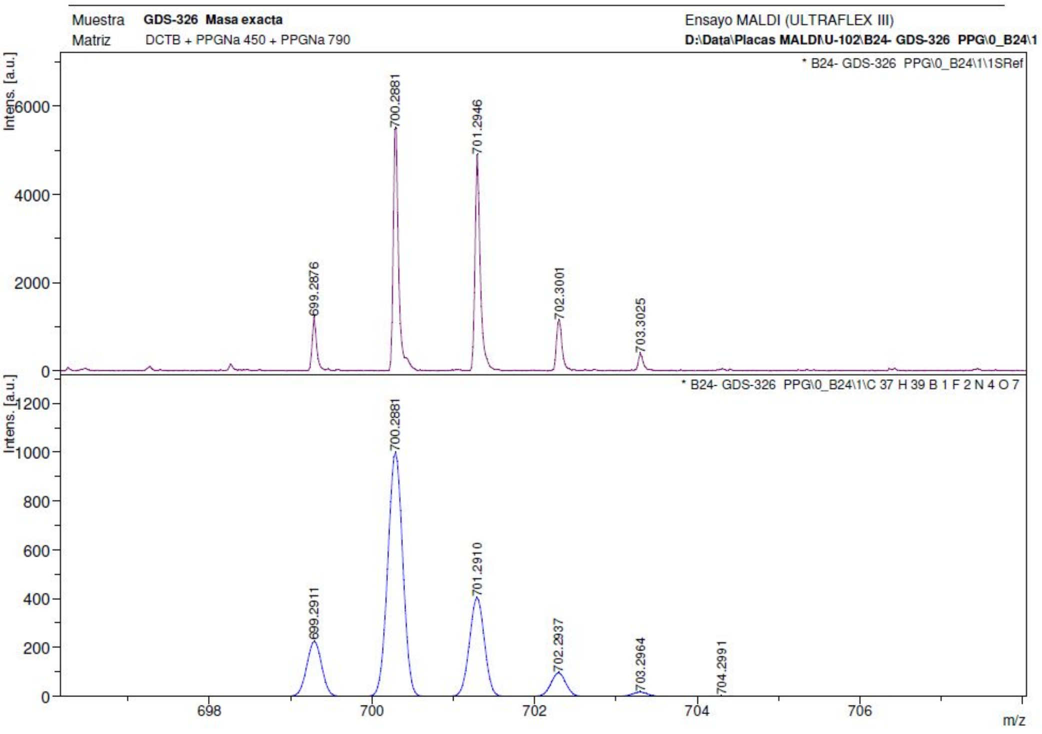

Figure S29. Experimental (top) and theoretical (bottom) isotopic patterns of the molecular ion of **3** shown in the MALDI-TOF mass spectrum.

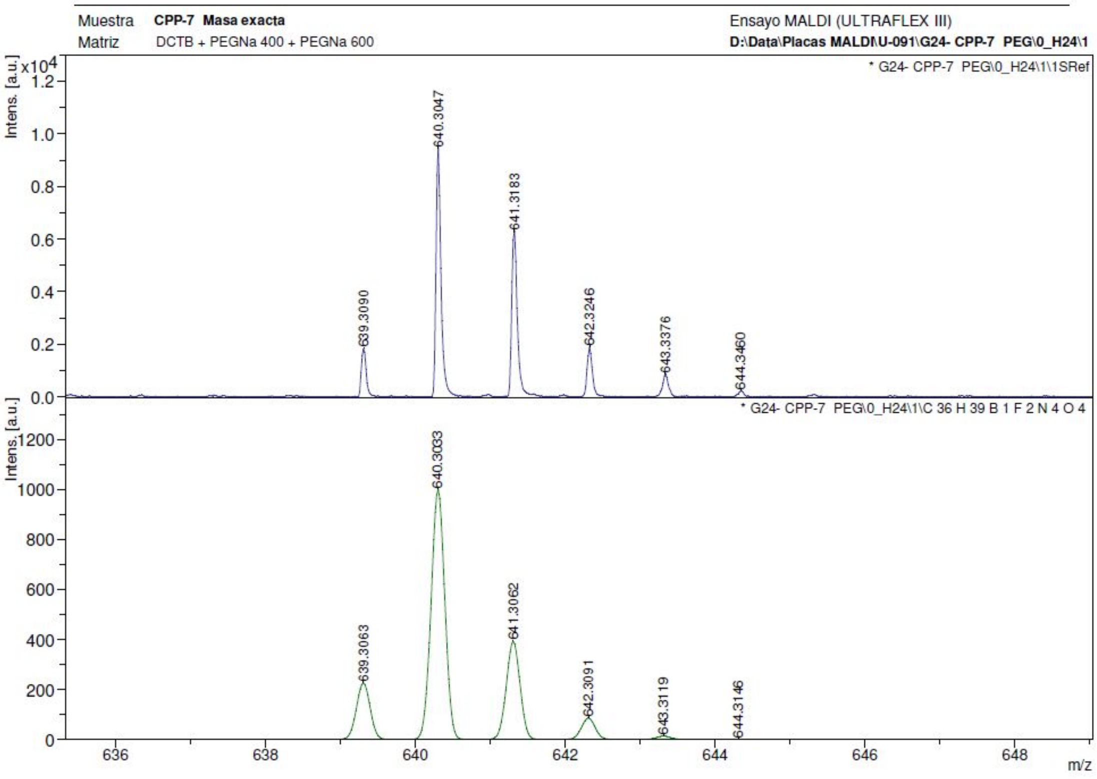

Figure S30. Experimental (top) and theoretical (bottom) isotopic patterns of the molecular ion of **4** shown in the MALDI-TOF mass spectrum.

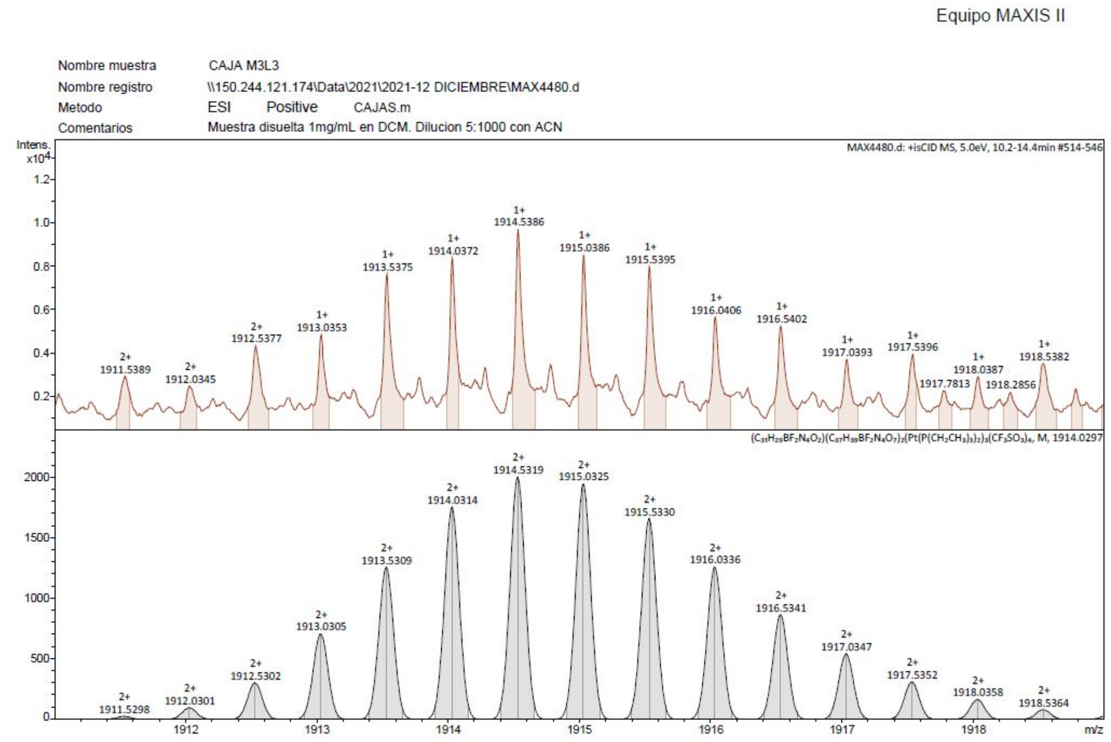

Figure S31. Experimental (top) and theoretical (bottom) isotopic patterns of the [M-2OTf$^-$]$^{2+}$ ion of **1** shown in the ESI mass spectrum.

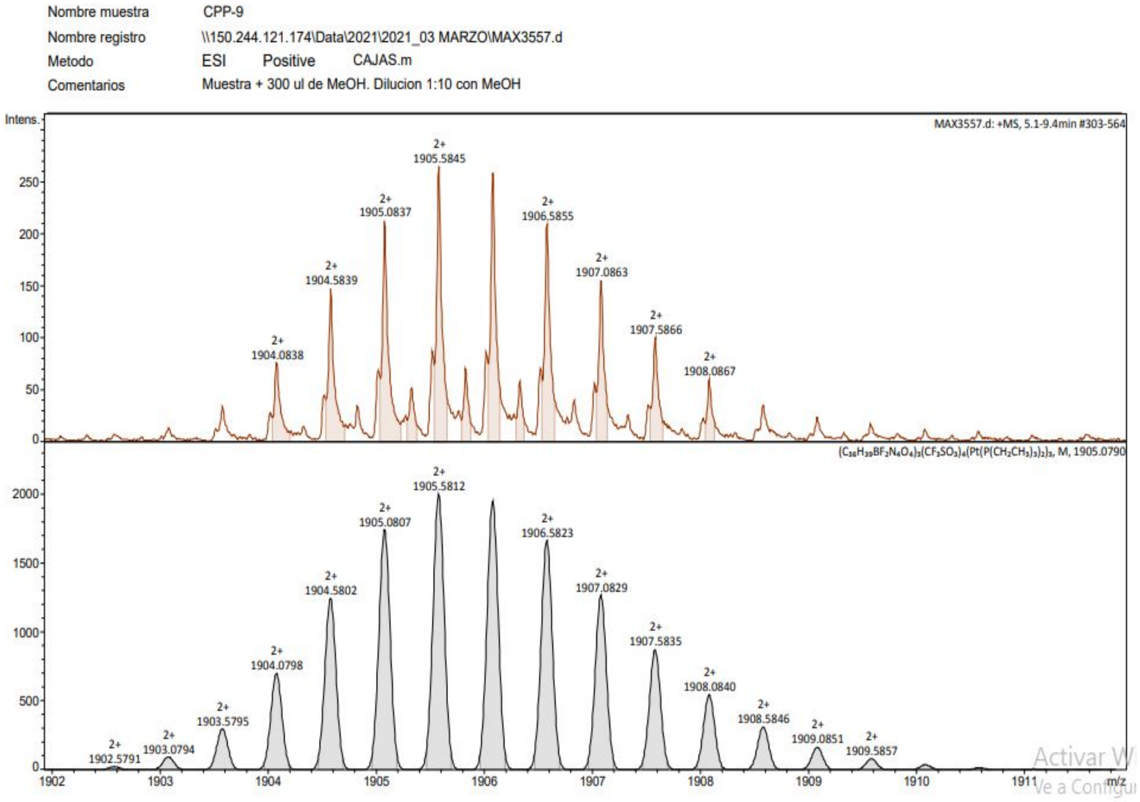

Figure S32. Experimental (top) and theoretical (bottom) isotopic patterns of the [M-2OTf$^-$]$^{2+}$ ion of **2** shown in the ESI mass spectrum.

4. HPLC chromatograms

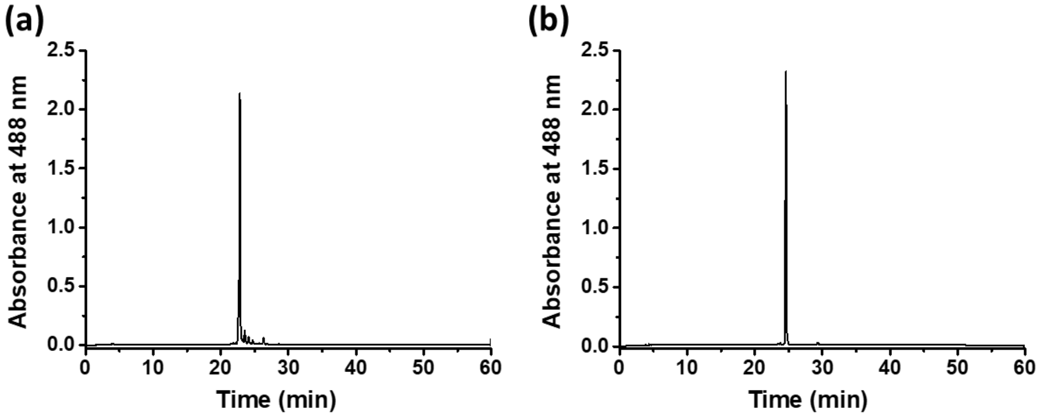

Figure S33. HPLC chromatograms of (a) **1** and (b) **2**.
